# Identification of Candidate Genes for Lint Percentage and Fiber Quality Through QTL Mapping and Transcriptome Analysis in an Allotetraploid Interspecific Cotton CSSLs Population

**DOI:** 10.3389/fpls.2022.882051

**Published:** 2022-04-29

**Authors:** Peng Yang, Xiaoting Sun, Xueying Liu, Wenwen Wang, Yongshui Hao, Lei Chen, Jun Liu, Hailun He, Taorui Zhang, Wanyu Bao, Yihua Tang, Xinran He, Mengya Ji, Kai Guo, Dexin Liu, Zhonghua Teng, Dajun Liu, Jian Zhang, Zhengsheng Zhang

**Affiliations:** College of Agronomy and Biotechnology, Southwest University, Chongqing, China

**Keywords:** *Gossypium barbadense*, transcriptome, quantitative trait loci, chromosome segment substitution lines, lint percentage, fiber quality

## Abstract

Upland cotton (*Gossypium hirsutum*) has long been an important fiber crop, but the narrow genetic diversity of modern *G. hirsutum* limits the potential for simultaneous improvement of yield and fiber quality. It is an effective approach to broaden the genetic base of *G. hirsutum* through introgression of novel alleles from *G. barbadense* with excellent fiber quality. In the present study, an interspecific chromosome segment substitution lines (CSSLs) population was established using *G. barbadense* cultivar Pima S-7 as the donor parent and *G. hirsutum* cultivar CCRI35 as the recipient parent. A total of 105 quantitative trait loci (QTL), including 85 QTL for fiber quality and 20 QTL for lint percentage (LP), were identified based on phenotypic data collected from four environments. Among these QTL, 25 stable QTL were detected in two or more environments, including four for LP, eleven for fiber length (FL), three for fiber strength (FS), six for fiber micronaire (FM), and one for fiber elongation (FE). Eleven QTL clusters were observed on nine chromosomes, of which seven QTL clusters harbored stable QTL. Moreover, eleven major QTL for fiber quality were verified through analysis of introgressed segments of the eight superior lines with the best comprehensive phenotypes. A total of 586 putative candidate genes were identified for 25 stable QTL associated with lint percentage and fiber quality through transcriptome analysis. Furthermore, three candidate genes for FL, *GH_A08G1681* (*GhSCPL40*), *GH_A12G2328* (*GhPBL19*), and *GH_D02G0370* (*GhHSP22.7*), and one candidate gene for FM, *GH_D05G1346* (*GhAPG*), were identified through RNA-Seq and qRT-PCR analysis. These results lay the foundation for understanding the molecular regulatory mechanism of fiber development and provide valuable information for marker-assisted selection (MAS) in cotton breeding.

## Introduction

Cotton (*Gossypium* spp.) fiber is one of the key raw materials used in the textile industry. Allotetraploid *G. hirsutum* and *G. barbadense* are the most important cultivated species, accounting for 95 and 2% of the total cotton yield worldwide, respectively (Liu et al., 2013). *G. hirsutum* has undergone a long period of domestication and selection for high-yield and fine-quality cotton. However, extensive domestication of this species has resulted in a narrow genetic basis and limited genetic diversity of the current *G. hirsutum* variety (Wendel et al., 2009). These characteristics of modern *G. hirsutum* cultivars are not advantageous for simultaneous improvement of yield and fiber quality. *G. barbadense* is well known for its extra-long-staple fibers with high strength and low micronaire, and it retains a high proportion of the diversity due to less intensive selection (Wendel et al., 2009). Whole-genome comparative analyses of *G. hirsutum* and *G. barbadense* revealed a large number of specific gene amplifications and structural variants between them (Hu et al., 2019). In particular, the specific expression of some genes related to fiber development may be an important reason for the excellent fiber quality of *G. barbadense*. Therefore, *G. barbadense* is a useful resource for fiber quality improvement in *G. hirsutum* breeding (Cao et al., 2015). Compared with traditional breeding methods, quantitative trait loci (QTL) mapping with molecular markers provides a powerful approach to transfer beneficial genes from *G. barbadense* to *G. hirsutum*.

Fiber yield and quality traits are complex quantitative traits controlled by multiple genes and affected by environments (Said et al., 2013). Identification of QTL for fiber yield and quality traits contributed to the improvement of fiber yield and quality in upland cotton. Up to now, a large number of QTL for fiber quality and yield traits have been identified in linkage studies (Said et al., 2013, 2015a,b). Moreover, with the rapid development of genome sequencing, genome-wide association studies (GWASs) have also identified many fiber quality QTL and yield QTL (Fang et al., 2017; Wang et al., 2017a; Ma et al., 2018b, 2021; Shen et al., 2019; Zhao et al., 2021). Meanwhile, application of chromosome segment substitution lines (CSSLs) in exploring complex genetic traits and accurately identifying QTL and genes in food and commercial crops has been widely studied (Balakrishnan et al., 2019), since the first set of tomato CSSLs was reported (Eshed and Zamir, 1994). However, most CSSLs reported in cotton are mainly developed with *G. hirsutum* genetic standard line TM-1 as the recurrent parent and *G. barbadense* line (such as Hai1, Hai 7,124, and 3–79) as the donor parent (Wang et al., 2012b; Zhang et al., 2016; Si et al., 2017; Balakrishnan et al., 2019), with fewer commercial cultivars as recurrent or donor parents (Deng et al., 2019; Shi et al., 2020). Moreover, most CSSLs populations ranged from 100 to 300 lines (Balakrishnan et al., 2019). The introgressed segments are difficult to cover the entire genome of genetic background. Therefore, it is necessary to establish larger populations of CSSLs and identify QTL candidate genes for yield and fiber quality using commercial cultivars as the recurrent parents or as the donor parents.

In the present study, a set of CSSLs was constructed using *G. barbadense* cultivar Pima S-7 as the donor parent and *G. hirsutum* cultivar CCRI35 as the recurrent parent. The CSSLs population was evaluated to dissect the genetic basis of lint percentage (LP) and fiber quality traits in multiple environments. In addition, RNA-Seq data and qRT-PCR were used to identify candidate genes for stable QTL and QTL clusters. The introgression lines with superior *G. barbadense* alleles and the environment-stable QTL identified can be used to improve fiber quality and yield of *G. hirsutum*.

## Materials and Methods

### Materials and Population Development

A set of CSSLs was constructed by crossing and backcrossing between donor parent Pima S-7 and recurrent parent CCRI35. Pima S-7 is a commercially grown *G. barbadense* cultivar from the USA, which has excellent fiber quality (Turcotte et al., 1992). CCRI35 is a *G. hirsutum* cultivar characterized by high-yield and disease-resistance and was released by the National Cotton Germplasm Resource Platform in China (Tan et al., 2015).

The F_1_ population was produced by crossing with CCRI35 as the female parent and Pima S-7 as the male parent in the summer of 2010 at the experimental station of Southwest University, Chongqing, China. The (CCRI35 x Pima S-7) F_1_ progeny were backcrossed with CCRI35 in the summer of 2011 in Chongqing to produce 200 BC_1_F_1_ plants. BC_2_F_1_ generation was produced by backcrossing BC_1_ progeny to CCRI35 in the summer of 2012 in Chongqing. Further, an advanced backcross generation of BC_3_F_1_ was obtained in 2013. A total of 200 BC_3_F_1_ lines were selfed and individually planted in Chongqing to produce BC_3_F_2_ in the summer of 2014. In addition, a total of 600 BC_3_F_2_ individual plants were planted in 2015, and fresh leaves of each plant were used for extraction of DNA samples. Individual plants that did not contain the introgressed chromosome segments of *G. barbadense* were eliminated through MAS screening. Through plant-to-row method, 562 BC_3_F_2:3_ family lines were planted in 2016 in Chongqing. BC_3_F_2:4_ family lines were planted in 2017 in Chongqing, and 562 BC_3_F_2:5_ lines were planted in 2018 in Chongqing (2018CQ) for phenotypic analysis and DNA extraction. BC_3_F_2:6_ family lines were grown for further evaluation in 2019 using the plant-to-row method in Chongqing (2019CQ) and Kuerle (Xinjiang Autonomous Region) (2019XJ). BC_3_F_2:7_ family lines were planted in 2020 in Chongqing (2020CQ) for phenotypic analysis. A plastic film covering and wide/narrow row spacing patterns were applied for 2019XJ. Row spacing alternation was 0.2 m and 0.6 m. A total of 15 plants of one genotype were grown in 5-m-long rows with a 0.7-m row spacing and an average plant spacing of 0.3 m for 2018CQ, 2019CQ, and 2020CQ. Standard field management was used for planting in each environment. A flow diagram showing the development process for the CSSLs population is presented in Supplementary Figure S1.

### Phenotypic Data Collection

All naturally opened bolls of BC_3_F_2:5_ individual plants of CSSLs were hand-harvested in 2018 in Chongqing. Thirty naturally opened bolls were picked up from each family line in the other environments (2019CQ, 2019XJ, and 2020CQ). The seed cotton was weighed and ginned to obtain LP data. Cotton fiber samples were sent to the Cotton Quality Supervision and Inspection Center of the Ministry of Agriculture of China for testing fiber quality with High Volume Instrument (HVI) 900 instrument (Uster® Hvispectrum, Spinlab, USA). The fiber quality traits included FL (mm), FS (cN/tex), FE (%), FM (unit), and fiber uniformity (FU, %).

### DNA Extraction and Genotype Detection

Cotton genomic DNA was extracted from fresh young leaves of the CSSLs and their parents at the seeding stage with the cetyltrimethylammonium bromide (CTAB) method reported by Zhang et al. (2005). A high-density genetic linkage map of the BC_1_ population [(CCRI35 × Pima S-7) × CCRI35] was previously constructed in our laboratory (Wang et al., 2017b). Simple sequence repeat (SSR) markers distributed on the genetic map were selected for genotype detection in the CSSLs. The average interval between two markers was approximately 10 centimorgans (cM). Physical locations of the markers were determined by aligning the marker sequence to the *G. hirsutum* TM-1 genome using the basic local alignment search program (BLAST); (Hu et al., 2019).

### Phenotypic and Genotypic Analysis

Descriptive statistical analysis of phenotypes was performed with Microsoft Excel 2016. The statistical values included maximum, minimum, mean, standard deviation, skewness, kurtosis, coefficients of variation (CV), and transgressive rate over the recurrent parent (TRORP). Analysis of variance (ANOVA), correlation analysis, and significance tests were performed with SPSS 18.0 software (SPSS, Chicago, Illinois, USA). GGT2.0 software (Van Berloo, 2008) was used to perform genotypic analysis of populations and calculations of chromosomal introgressed segments (including background recovery rate of the CSSLs, the number and length of introgressed segments).

### Identification of QTL and QTL Clusters

Multiple-QTL model (MQM) of MapQTL6.0 software (Van Ooijen, 2009) was used to identify QTL for lint percentage and fiber quality traits. A threshold of log of odds ratio (LOD) ≥ 2.0 was used to claim a putative QTL as suggested by Lander and Botstein (1989). Negative additive effects indicated that CCRI35 alleles increased the phenotypic trait values, and positive scores indicated that Pima S-7 alleles increased the phenotypic trait values. The QTL nomenclature was presented as follows: q + trait abbreviation + chromosome number + QTL number (Tang et al., 2015). The same QTL was defined as a QTL with overlapping confidence intervals for the same trait identified in different environments and with the same direction of additive effect (Shao et al., 2014; Ma et al., 2020). QTL identified in at least two generations or environments were considered to be stable QTL (Liu et al., 2017). If a chromosomal interval contained more than three or more QTL for multiple traits, and the confidence intervals of all QTL had an overlapping interval, these QTL formed a QTL cluster (Rong et al., 2007). The overlapping confidence intervals of these QTL were regarded as the confidence intervals of the QTL clusters.

### RNA Extraction, Library Construction, and Sequencing

Ovules with fiber were collected from CCRI35 and Pima S-7 at 0 days post-anthesis (DPA). Their fibers were collected at 8, 18, 25, and 32 DPA. Three biological replicates of tissue samples were collected at each developmental stage from both parents, which were planted in the winter of 2020 in Sanya, Hainan, China. Total RNA extraction of samples was performed using plant RNA extraction kit (Aidlab, Beijing, China) following the manufacturer’s instructions. Agilent 2,100 Bioanalyzer (Agilent Technologies, Palo Alto, CA, USA) was used to assess RNA quality, and RNA quality was detected using RNase-free agarose gel electrophoresis. High-quality deep sequencing was performed at the Gene Denovo Biotechnology Co. (Guangzhou, China) using Illumina NovaSeq 6,000 system. Gene expression level was calculated using fragments per kilobase of exon model per million mapped reads (FPKM). Genes with the parameter of false discovery rate (FDR) below 0.05 and absolute fold change ≥2 were considered differentially expressed genes (DEGs). The raw RNA-Seq data were deposited in NCBI Sequence Read Archive under the accession number PRJNA809429.

### Candidate Gene Identification and Annotation

Genes expressed (FPKM≥1) at one or more time points and located in the QTL or QTL clusters confidence intervals were considered as candidate genes. The positions of the two flanking markers determined the candidate regions. Genes located in the candidate regions were analyzed with TM-1 genome data as reference (Hu et al., 2019). All candidate genes were mapped to Gene Ontology (GO) terms in the GO database^1^ and Kyoto Encyclopedia of Genes and Genomes (KEGG) database^2^ to explore potential biological processes and metabolic pathways related to the genes. Expression profiles of candidate genes were analyzed and visualized using Omicsmart tools.^3^

### Weighted Gene Co-expression Network Analysis (WGCNA)

Co-expression networks were built with WGCNA function in Omicsmart (see footnoet 3). These modules were created with default parameters of the automated network build function block. Genes with high intramodular connectivity (K.in) values were identified as hub genes that may have important functions. Genes with low module correlation degree (MM) values (MM < 0.8) and K.in values within the module were filtered out to select putative candidate genes.

### Gene Expression Analysis by qRT-PCR

High-quality RNA was reverse-transcribed using the PrimeScript™ RT reagent Kit with gDNA Eraser (TaKaRa, Dalian, China). cDNA samples were used for quantitative real-time PCR (qRT-PCR) in a total volume of 20 μl using Hieff® qPCR SYBR® Green Master Mix (Yeasen, China). The real-time qTOWER 3 system (Analytik Jena, Germany) was used to perform qRT-PCR. *GH_A11G2385* (*GhActin7*) was used as the internal control. Relative expression level of candidate genes was calculated using the 2^-ΔΔCt^ method (Livak and Schmittgen, 2001).

## Results

### Phenotypic Performance of the CSSLs Population

Descriptive statistics of phenotypic traits of the CSSLs population are presented in Supplementary Table S1. The mean values of LP and FL were slightly lower relative to those of CCRI35. The mean values of FS were higher compared with those of CCRI35, and the mean values for FU, FM, and FE were similar to those of the recurrent parent CCRI35. Transgressive segregation was observed for all traits. The TRORP for lint percentage and fiber quality for CSSLs ranged from 3.81 to 61.05% and 6.56 to 90.02%, respectively. The large ranges of traits indicated the extensive genetic variation in the CSSLs population. Variation of FM was the highest among the six traits, followed by LP, whereas FU and FE showed the lowest variation.

The absolute skewness of all traits in all environments was less than one, thus following a normal distribution (Supplementary Table S1; Supplementary Figure S2). The results of ANOVA showed that genotype and environmental factors significantly affected all traits (P < 0.001) (Supplementary Table S2). Correlation analysis of the CSSLs population clearly showed the degree of correlation between the traits (Supplementary Table S3; Supplementary Figure S3). Seven paired traits (LP and FM, FL and FU/FS/FE, FU and FS/FE, and FS and FE) showed significant positive correlations in multiple environments. Four paired traits (LP and FL/FS, FL and FM, and FS and FM) showed significant negative correlations in multiple environments. Moreover, four paired traits (LP and FU/FE, FM and FU/FE) showed no or weak positive or negative correlations in multiple environments (Supplementary Table S3; Supplementary Figure S3).

### Genotypic Analysis of CSSLs Population

The 489 SSR markers evenly distributed on 26 chromosomes, with an average of 19 markers per chromosome (Supplementary Tables S4, S5). The total genetic distance of CSSLs covered 3833.61 cM, with an average marker interval of 7.92 cM, accounting for 99.2% of the whole genetic map.

**Figure 1:**
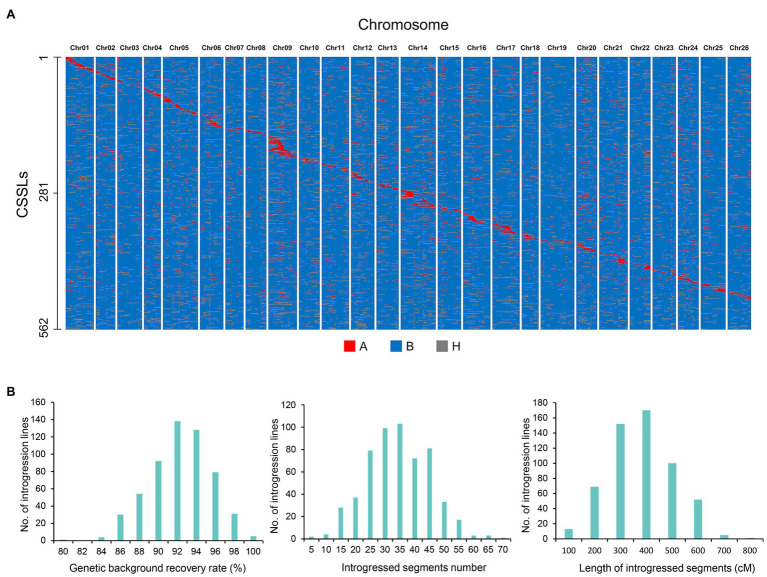
Graphical representation of genotypes and chromosome introgressed segment calculations of the CSSLs population. **(A)** Distribution of introgressed segments in the CSSLs on the 26 chromosomes. (A), Homozygous introgressed segments (donor parent Pima S-7); B, genetic background (recurrent parent CCRI35); H, heterozygous introgressed segments. **(B)** Genetic background recovery rate, number and length of introgressed segments in the CSSLs population.

The CSSLs population comprised of 562 lines with donor parents, and the introgressed Pima S-7 segments covered higher proportion of the genome (Figure 1A). The number of introgressed segments ranged from 1 to 70, and the length of introgressed Pima S-7 segments ranged from 200 cM to 500 cM in most lines, with an average length of 332.3 cM. The maximum length of introgressed segments from Pima S-7 in each line was 766.7 cM, whereas the minimum length was 3.8 cM. The rate of background recovery in the CSSLs ranged from 80 to 99.9%, with an average of 91.3%. Lines with more than 90% genetic background recovery ratio accounted for 67.8% of the total CSSLs (Figure 1B).

### QTL Identification for Lint Percentage and Fiber Quality Traits

**Figure 2:**
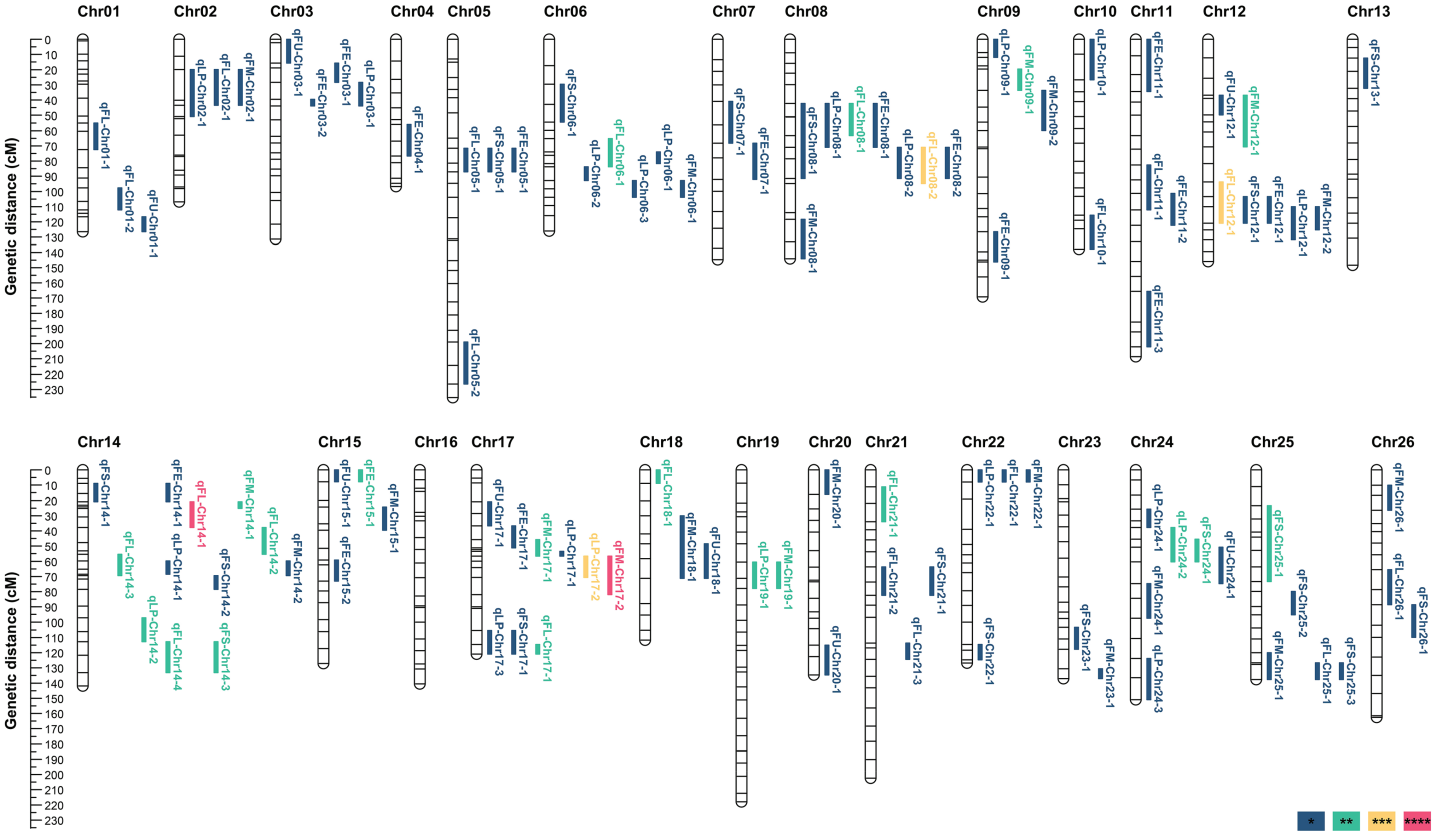
QTL for lint percentage and fiber quality traits identified across four environments. *, **, ***, and **** indicate that QTL were detected in one environment, two environments, three environments, and four environments, respectively.

A total of 105 QTL (49 in A_t_ and 56 in D_t_) were identified, including 20 QTL (10 in A_t_ and 10 in D_t_) for lint percentage and 85 QTL (39 in A_t_ and 46 in D_t_) for fiber quality. Out of the 105 QTL, 25 stable QTL were identified in two to four environments, including four for LP, eleven for FL, three for FS, six for FM, and one for FE (Table 1; Supplementary Table S6). These QTL mainly distributed on four chromosomes, including seven on Chr12, eight on Chr08, nine on Chr17, and twelve on Chr14 (Supplementary Table S6; Figure 2; Supplementary Figure S4).

**Table 1:** Distribution of stable QTL on chromosomes for each trait.

QTL^a^	Chromosome	No.^b^	Start (bp)	End (bp)
*qLP-Chr14-2*	Chr14	2	64,623,643	67,444,656
*qLP-Chr17-2*	Chr17	3	28,380,381	48,355,755
*qLP-Chr19-1*	Chr19	2	10,553,151	17,652,436
*qLP-Chr24-2*	Chr24	2	5,222,533	11,851,786
*qFL-Chr06-1*	Chr06	2	28,892,690	118,434,365
*qFL-Chr08-1*	Chr08	2	5,646,877	79,354,581
*qFL-Chr08-2*	Chr08	3	91,912,709	114,328,960
*qFL-Chr12-1*	Chr12	3	93,334,983	103,465,008
*qFL-Chr14-1*	Chr14	4	2,685,200	4,778,807
*qFL-Chr14-2*	Chr14	2	4,778,807	7,594,977
*qFL-Chr14-3*	Chr14	2	7,594,977	54,299,973
*qFL-Chr14-4*	Chr14	2	67,066,803	68,244,966
*qFL-Chr17-1*	Chr17	2	52,679,437	53,779,566
*qFL-Chr18-1*	Chr18	2	135,110	1,853,139
*qFL-Chr21-1*	Chr21	2	1,409,800	3,677,982
*qFS-Chr14-3*	Chr14	2	67,066,803	68,244,966
*qFS-Chr24-1*	Chr24	2	6,654,747	11,851,786
*qFS-Chr25-1*	Chr25	2	2,969,257	43,008,423
*qFM-Chr09-1*	Chr09	2	6,881,842	8,284,577
*qFM-Chr12-1*	Chr12	2	4,543,472	83,872,431
*qFM-Chr14-1*	Chr14	2	2,685,200	4,363,465
*qFM-Chr17-1*	Chr17	2	3,280,764	26,177,027
*qFM-Chr17-2*	Chr17	4	28,380,381	49,203,384
*qFM-Chr19-1*	Chr19	2	10,553,151	17,652,436
*qFE-Chr15-1*	Chr15	2	239,251	1,570,620

a *LP, lint percentage; FL, fiber length; FS, fiber strength; FM, fiber micronaire; FE, fiber elongation*.

b *Number of environments*.

Twenty QTL for LP were identified on 12 chromosomes. Among these QTL, 12 favorable alleles were contributed by CCRI35, whereas eight were derived from Pima S-7. Three QTL (*qLP-Chr14-2*, *qLP-Chr19-1*, and *qLP-Chr24-2*) were identified across two environments and one QTL (*qLP-Chr17-2*) was identified in three environments.

Twenty-three QTL for FL were identified on 15 chromosomes. Fourteen favorable alleles were contributed by Pima S-7, whereas the other nine favorable alleles were derived from CCRI35. Eleven stable QTL for FL were detected. Among these stable QTL, two QTL (*qFL-Chr08-2* and *qFL-Chr12-1*) were identified in three environments and one QTL (*qFL-Chr14-1*) was detected in four environments.

Eight QTL for FU were identified on eight chromosomes. All QTL were detected in only one environment. Three favorable alleles were contributed by Pima S-7, whereas five favorable alleles originated from CCRI35.

Eighteen QTL for FS were identified on 14 chromosomes. Among these QTL, 12 favorable alleles were contributed by Pima S-7, whereas six favorable alleles were derived from CCRI35. Three QTL (*qFS-Chr14-3*, *qFS-Chr24-1*, and *qFS-Chr25-1*) were detected in two environments.

Twenty QTL for FM were mapped on 16 chromosomes. Among these FM-QTL, 13 favorable alleles decreasing FM value were contributed by Pima S-7, whereas the others came from CCRI35. One QTL (*qFM-Chr17-2*) was identified in four environments and five QTL (*qFM-Chr09-1*, *qFM-Chr12-1*, *qFM-Chr14-1*, *qFM-Chr17-1*, and *qFM-Chr19-1*) were identified in two environments.

Sixteen QTL for FE were mapped on 11 chromosomes. Ten favorable alleles were derived from Pima S-7, whereas six were contributed by CCRI35. Only one stable QTL (*qFE-Chr15-1*) was identified in two environments.

### QTL Cluster Analysis

Five and six QTL clusters were distributed on the At and Dt subgenomes, respectively (Table 2). Out of the 11 QTL clusters, seven harbored at least one stable QTL. Cluster-Chr08-1 harbored three QTL and included one stable QTL, *qFL-Chr08-1*. Cluster-Chr08-2 harbored four QTL, and the stable QTL, *qFL-Chr08-2*, was detected across three environments. Two QTL clusters (Cluster-Chr14-1 and Cluster-Chr14-2) were identified on Chr14. Cluster-Chr14-1 harbored three QTL with one stable QTL (*qFL-Chr14-3*), and Cluster-Chr14-2 harbored three stable QTL (*qLP-Chr14-2*, *qFL-Chr14-4*, and *qFS-Chr14-3*). Cluster-Chr12-1 harbored five QTL and included one stable QTL (*qFL-Chr12-1*). Cluster-Chr24-1 comprised of three QTL and included two stable QTL, *qLP-Chr24-2* and *qFS-Chr24-1*.

**Table 2:** QTL clusters for lint percentage and fiber quality traits identified in the CSSLs across multiple environments.

Cluster	Physical distance interval (bp)	No. of genes	QTL
Cluster-Chr02-1	3,105,422–7,656,327	290	qLP-Chr02-1(+), qFL-Chr02-1(−)^b^, qFM-Chr02-1(+)^b^
Cluster-Chr05-1	13,051,682–17,383,572	424	qFL-Chr05-1(+)^b^, qFS-Chr05-1(+)^b^, qFE-Chr05-1(+)^b^
Cluster-Chr08-1	5,646,877–79,354,581	775	qLP-Chr08-1(+)^b^, qFL-Chr08-1(−)^ab^, qFE-Chr08-1(−)^b^
Cluster-Chr08-2	91,912,709–108,725,736	367	qLP-Chr08-2(+), qFL-Chr08-2(−)^ab^, qFS-Chr08-1(−)^b^, qFE-Chr08-2(−)^b^
Cluster-Chr12-1	101,133,695–103,465,008	216	qLP-Chr12-1(−)^b^, qFL-Chr12-1(+)^a^, qFS-Chr12-1(+), qFM-Chr12-2(−), qFE-Chr12-1(+)
Cluster-Chr14-1	9,015,027–51,535,517	876	qLP-Chr14-1(−)^b^, qFL-Chr14-3(+)^ab^, qFM-Chr14-2(−)
Cluster-Chr14-2	67,066,803–67,444,656	52	qLP-Chr14-2(−)^a^, qFL-Chr14-4(+)^ab^, qFS-Chr14-3(+)^a^
Cluster-Chr17-1	51,040,747–53,779,566	205	qLP-Chr17-3(+), qFL-Chr17-1(−)^a^, qFS-Chr17-1(−)
Cluster-Chr22-1	611,722–2,149,127	130	qLP-Chr22-1(+), qFL-Chr22-1(−)^b^, qFM-Chr22-1(+)
Cluster-Chr24-1	9,643,920–11,851,786	72	qLP-Chr24-2(−)^a^, qFS-Chr24-1(+)^ab^, qFU-Chr24-1(+)^b^
Cluster-Chr25-1	64,007,912–64,899,957	107	qFL-Chr25-1(−)^b^, qFS-Chr25-3(−), qFM-Chr25-1(+)^b^

a *Stable QTL*.

b *Common QTL*.

*(+) Positive additive effects indicate that Pima S-7 alleles increased the phenotypic value*.

*(−) Negative additive effects indicate that CCRI35 alleles increased the phenotypic value*.

### Transcriptome Analysis of Fiber Development

Transcriptome analysis was conducted on tissue samples from CCRI35 and Pima S-7 at five time-points during fiber development. RNA-Seq experiments yielded 38–61 million clean reads per sample, which were used for further analyses. Approximately 95.58 to 98.18% of the clean reads were mapped to the TM-1 reference genome (Supplementary Table S7). The global gene expression profile is presented in Supplementary Figure S5. The results indicated sufficient coverage of the transcriptome during fiber development of the two allotetraploid cottons.

The DEGs between CCRI35 and Pima S-7 were grouped into up-regulated and down-regulated genes. Up-regulated genes referred to genes that were expressed at higher levels in Pima S-7 compared with the expression level in CCRI35, whereas the opposite was true for down-regulated genes. The number of up-regulated genes was less compared with the number of down-regulated genes at all developmental stages (Supplementary Figure S6). These DEGs were grouped into ten categories, including five categories for up-regulated genes and five categories for down-regulated genes. GO enrichment analysis was then performed based on DEGs in each category to identify enriched terms in the biological processes, cellular components, and molecular function categories (Supplementary Table S8). Genes involved in the intrinsic component of membrane, catalytic activity, and membrane part were significantly enriched in nine, eight, and seven categories, respectively. This result implies that these DEGs played an important role in the fiber quality differences between *G. barbadense* and *G. hirsutum*.

### Candidate Gene Annotation

The candidate genes within the identified QTL intervals are presented in Supplementary Table S9. The candidate genes were annotated through GO and KEGG analyses (Supplementary Tables S10, S11). Nucleic acid binding, organic substance metabolic process, mRNA processing, inositol phosphate phosphatase activity, hydro-lyase activity, and cellular catabolic process were the most significantly enriched GO terms associated with candidate genes for LP, FL, FU, FS, FM, and FE. Metabolic process and biosynthesis of secondary metabolites were the top two most enriched KEGG pathways associated with the highest numbers of candidate genes for each trait. Expression level of the candidate genes in QTL clusters during fiber development is presented in Supplementary Table S12. A total of 2,186 candidate genes were detected within the confidence interval of the QTL clusters. These candidate genes were annotated through GO and KEGG pathway analyses (Supplementary Tables S13, S14). A total of 1,131 candidate genes could be annotated with a total of 1722 GO terms, and some candidate genes could be annotated with more than one GO term. Moreover, a total of 535 candidate genes were annotated with 112 KEGG pathways.

### Identification of Putative Candidate Genes in Gene Co-expression Network Modules

WGCNA was used to generate co-expression networks for 14,763 genes within QTL intervals to identify putative candidate genes associated with fiber quality. A total of 20 modules were identified by WGCNA. Among these modules, 13 modules were significantly related to an individual developmental stage of CCRI35 and Pima S-7 or were specifically correlated with specific fiber developmental stages based on the coefficient between modules and tissues (Supplementary Table S15; Supplementary Figure S7). Five modules were significantly correlated with high-quality fiber (Pima S-7), and eight modules were significantly correlated with medium-quality fiber (CCRI35). The candidate genes within the stable QTL intervals were selected for further analysis. The top-ranked genes with the highest connectivity in each of the 13 modules were selected as putative candidate genes based on the K.in values and MM values of each gene (Supplementary Table S16). A total of 586 putative candidate genes for 25 stable QTL were identified.

### Prediction of Candidate Genes of the Stable QTL

We investigated candidate genes for QTL with smaller physical intervals. Furthermore, RNA-Seq analysis was performed to explore expression profiles of candidate genes at different fiber developmental stages of CCRI35 and Pima S-7, and the results were validated with qRT-PCR (Supplementary Table S17). Four candidate genes associated with stable QTL (3 for FL, 1 for FM) were finally identified.

**Figure 3:**
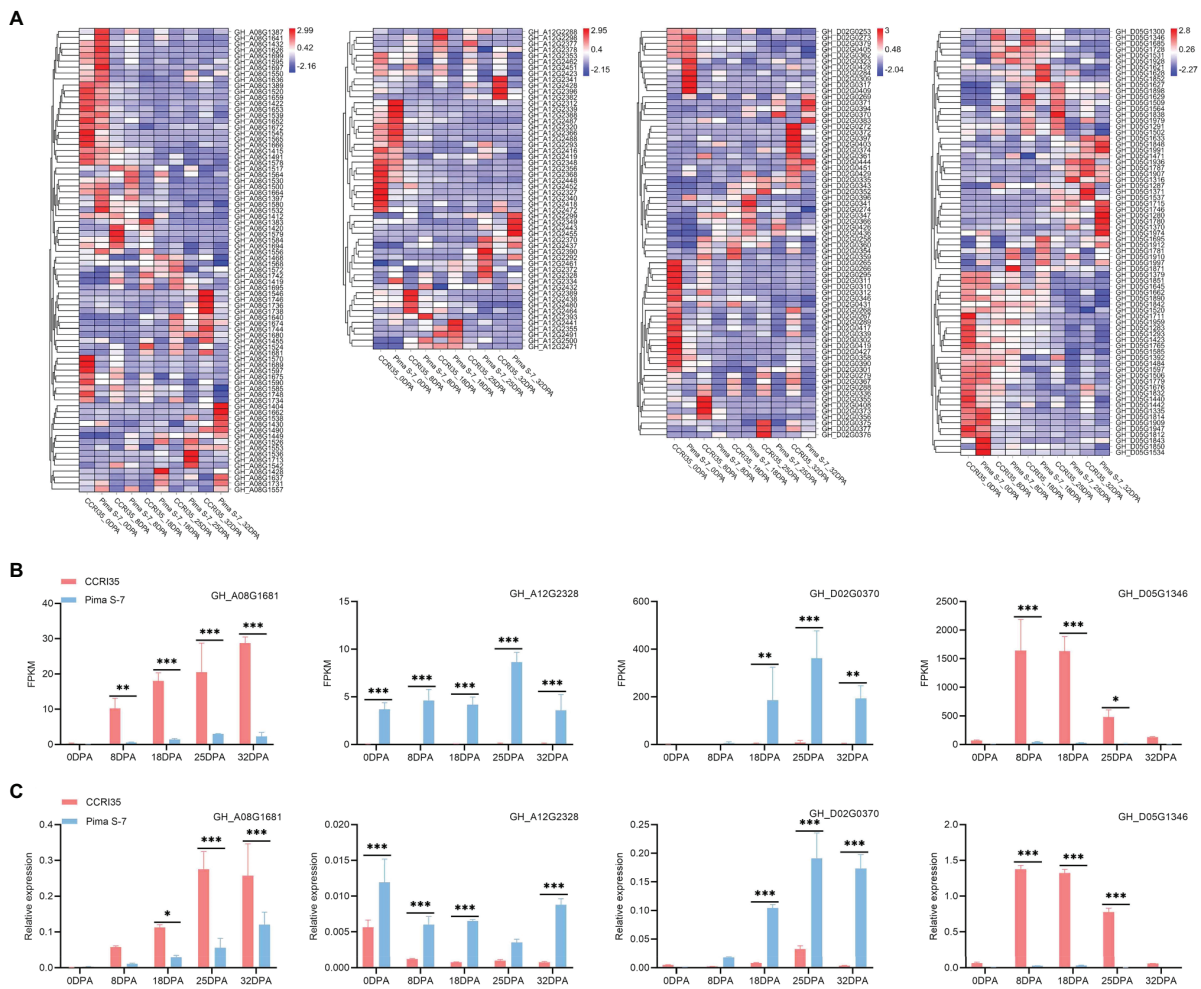
Expression profiles of fiber-related genes at different development stages. 0 DPA: mixture of ovule and fiber; 8, 18, 25, and 32 DPA: fiber. **(A)** Heat map showing expression profiles of candidate genes for stable QTL (*qFL-Chr08-2, qFL-Chr12-1, qFL-Chr14-1*, and *qFM-Chr19-1*) at different fiber development stages. **(B)** FPKM of *GH_A08G1681*, *GH_A12G2328*, *GH_D02G0370*, and *GH_D05G1346* at different developmental stages of the two varieties. **(C)** Expression level of the four candidate genes associated with fiber as determined by qRT-PCR. *, **, and ***: significant levels at 0.5, 0.01, and 0.001, respectively.

*qFL-Chr08-2* detected in three environments was overlapped with *qLP-Chr08-2*, *qFS-Chr08-1*, and *qFE-Chr08-2* in Cluster- Chr08-2 (Table 1; Supplementary Table S6). The overlapping confidence interval of *qFL-Chr08-2* corresponded to a 16,813,027-bp genome sequence (from 91,912,709 bp to 108,725,736 bp) in the *G. hirsutum* reference genome. The physical interval harbored 367 annotated genes (*GH_A08G1383*-*GH_A08G1749*) and 78 DEGs. One DEG (*GH_A08G1681*) was relatively significantly up-regulated in CCRI35 compared with the expression in Pima S-7 from 8 to 32 DPA during fiber development (Figures 3A,B). In addition, *GH_A08G1681* was identified in the dark red module by WGCAN and was a putative candidate gene in *qFL-Chr08-2* (Supplementary Table S16). The qRT-PCR result indicated *GH_A08G1681* was relatively highly expressed at fiber development stages (18–32 DPA) in CCRI35 than in Pima S-7 (Figure 3C). The RNA-Seq data revealed one nonsynonymous SNP mutation in exon 5 of *GH_A08G1681* (CCRI35 to Pima S-7, exon5: c.G1071T: p.R357S) and one SNP mutation in the upstream of *GH_A08G1681* (CCRI35/Pima S-7, 105,733,855: G/A) (Supplementary Table S18).

*qFL-Chr12-1* detected in three environments was overlapped with *qLP-Chr12-1*, *qFS-Chr12-1*, *qFM-Chr12-2*, and *qFE-Chr12-1* in Cluster-Chr12-1 (Table 1; Supplementary Table S6). The overlapping confidence interval of *qFL-Chr12-1* corresponded to a 2,331,313-bp genome sequence (from 101,133,695 bp to 103,465,008 bp) in the *G. hirsutum* reference genome. The physical interval harbored 216 annotated genes (*GH_A12G2287*-*GH_A12G2502*) and 54 DEGs. Among these DEGs, *GH_A12G2328* was significantly highly expressed in Pima S-7 than in CCRI35 from 0 to 32 DPA during fiber development (Figures 3A,B). *GH_A12G2328* was identified in the black red module by WGCAN and was a putative candidate gene in *qFL-Chr12-1* (Supplementary Table S16). The qRT-PCR result indicated that *GH_A12G2328* had significantly higher expression in Pima S-7 than in CCRI35 during fiber development period except at 25 DPA (Figure 3C). The RNA-Seq data revealed one nonsynonymous SNP mutation in exon 3 of *GH_A12G2328* (CCRI35 to Pima S-7, exon3: c.T254C: p.L85P) and one nonsynonymous SNP mutation in exon 4 of *GH_A12G2328* (CCRI35 to Pima S-7,exon4: c.A494G: p.D165G) (Supplementary Table S18).

*qFL-Chr14-1* detected in four environments (Supplementary Table S6). The confidence interval of *qFL-Chr14-1* corresponded to a 2,093,607-bp genome sequence (from 2,685,200 bp to 4,778,807 bp) in the *G. hirsutum* reference genome. The physical interval harbored 145 annotated genes (*GH_D02G0244*-*GH_D02G0388*) and 69 DEGs. One DEG (*GH_D02G0370*) showed significantly higher expression in Pima S-7 than in CCRI35 during fiber development from 18 to 32 DPA (Figures 3A,B). *GH_D02G0370* was identified in the dark turquoise module by WGCAN and was a putative candidate gene in *qFL-Chr14-1* (Supplementary Table S16). The qRT-PCR result indicated that the expression of *GH_D02G0370* was significantly up-regulated in Pima S-7 than in CCRI35 from 18 to 32 DPA during fiber development, which was consistent with RNA-Seq results (Figure 3). The RNA-Seq data revealed three nonsynonymous SNP mutations in exon 1 of *GH_D02G0370* (CCRI35 to Pima S-7, exon1: c.A146C: p.N49T, exon1: c.A365T: p.N122I, exon1: c.C489G: p.H163Q) and five SNP mutations in the upstream of *GH_D02G0370* (CCRI35/Pima S-7, 4,625,905: C/A, 4625924: A/C, 4625933: T/C, 4626041: A/G, 4626063: G/A) (Supplementary Table S18).

*qFM-Chr19-1* detected in two environments was overlapped with *qLP-Chr19-1* (Supplementary Table S6). A total of 794 annotated genes were identified within the confidence interval of *qFM-Chr19-1*. Out of the 794 genes, 346 genes were expressed during fiber development, including 72 DEGs (Figure 3A). One DEG (*GH_D05G1346*) showed significantly higher expression in CCRI35 than in Pima S-7 from 8 to 25 DPA during fiber development (Figures 3A,B). In addition, *GH_D05G1346* was identified in the royal blue module by WGCAN and was a putative candidate gene in *qFM-Chr19-1*(Supplementary Table S16). The qRT-PCR result indicated that *GH_D05G1346* had significantly higher expression in CCRI35 than in Pima S-7 from 8 to 25 DPA during fiber development, which was consistent with the RNA-Seq results (Figure 3). The RNA-Seq data revealed one nonsynonymous SNP mutation in exon 5 of *GH_D05G1346* (CCRI35 to Pima S-7, exon5: c.T1038A: p.D346E) and one SNP mutation in the upstream of *GH_D05G1346* (CCRI35/Pima S-7, 11,208,632; A/T) (Supplementary Table S18).

## Discussion

### Comparison of QTL With Previous Reports

To determine whether the QTL in the present study were common QTL, we compared our results with previous linkage and association studies. A total of 57 QTL shared the same or overlapping confidence intervals with QTL identified in previous studies (Supplementary Tables S19, S20). Out of the 57 common QTL, 29 QTL were identified in previous GWAS (Fang et al., 2017; Ma et al., 2018b, 2021; Zhao et al., 2021). Fifteen QTL (*qFL-Chr05-1*, *qFL-Chr06-1*, *qFL-Chr10-1*, *qFM-Chr09-2*, *qFM-Chr14-1*, *qFM-Chr24-1*, *qFS-Chr07-1*, *qFS-Chr23-1*, *qFS-Chr24-1*, *qFS-Chr25-1*, *qFS-Chr26-1*, *qFU-Chr24-1*, *qLP-Chr12-1*, *qLP-Chr17-2*, and *qLP-Chr24-1*) identified in the present study have been reported in multiple studies (Wang et al., 2006, 2012a, 2016; Sun et al., 2012; Shao et al., 2014; Cao et al., 2015; Liang et al., 2015; Shi et al., 2015, 2019; Zhang et al., 2015; Fang et al., 2017; Si et al., 2017; Diouf et al., 2018; Ma et al., 2018b, 2021; Deng et al., 2019; Feng et al., 2019; Li et al., 2019a,b; Zhao et al., 2021). These common QTL would be valuable for *G. hirsutum* breeding.

### *G. barbadense* Has More Favorable Alleles for Fiber Quality QTL

Previously studies reported that more QTL controlling fiber quality traits are mainly located on the D_t_ subgenome (Paterson et al., 2003; Rong et al., 2007; Said et al., 2013; Shi et al., 2020). The present study identified 39 and 46 QTL for fiber quality were distributed on A_t_ and D_t_ subgenomes, respectively. More fiber quality QTL distributed on the D_t_ subgenome compared with the number on the A_t_ subgenome, which was consistent with the previous studies. Moreover, favorable alleles for fiber quality QTL were mainly from *G. barbadense* (52 from *G. barbadense* and 33 from *G. hirsutum*; Table 2).

### Excellent Introgression Lines With More Favorable QTL Alleles for Fiber Quality

Full utilization of genetic resources of *G. barbadense* with excellent fiber quality should be considered to promote genetic enhancement of widely cultivated *G. hirsutum* varieties (Zhu et al., 2020). Eight superior lines with the best comprehensive LP, FL, FS, and FM phenotypes in four environments were identified in the CSSLs population (Table 3).

**Table 3:** Phenotypic means of the eight lines with excellent fiber quality in four environments.

Line ID	LP (%) ± SD	FL (mm) ± SD	FS (cN/tex) ± SD	FM (unit) ± SD	FU (%) ± SD	FE (%) ± SD
CSSL-23	35.41 ± 1.84	31.53 ± 2.63	36.90 ± 3.24	3.87 ± 0.12	84.27 ± 0.90	6.80 ± 0.17
CSSL-53	36.26 ± 2.86	31.60 ± 1.06	35.55 ± 3.83	4.15 ± 0.45	84.45 ± 2.05	6.80 ± 0.08
CSSL-277	37.60 ± 2.28	31.50 ± 0.77	35.60 ± 2.26	4.03 ± 0.05	85.25 ± 1.14	6.75 ± 0.13
CSSL-292	35.71 ± 4.72	32.73 ± 1.55	33.38 ± 0.52	3.95 ± 0.49	85.10 ± 1.07	6.85 ± 0.13
CSSL-295	34.30 ± 3.21	32.13 ± 2.06	35.60 ± 4.30	4.23 ± 0.42	84.17 ± 0.87	6.83 ± 0.15
CSSL-364	35.39 ± 2.74	31.60 ± 1.00	37.75 ± 4.23	4.33 ± 0.62	85.15 ± 0.42	6.85 ± 0.06
CSSL-523	36.28 ± 2.17	32.00 ± 2.54	37.10 ± 2.17	4.17 ± 0.12	85.60 ± 0.78	6.83 ± 0.06
CSSL-552	36.62 ± 2.62	32.20 ± 1.21	37.18 ± 4.87	4.48 ± 0.51	84.65 ± 0.64	6.85 ± 0.06
CCRI35	39.80 ± 2.41	30.35 ± 0.81	31.60 ± 1.33	4.53 ± 0.21	84.35 ± 1.52	6.75 ± 0.13

*ID, identity; SD, standard deviation; LP, lint percentage; FL, fiber length; FS, fiber strength; FM, fiber micronaire; FU fiber uniformity; FE, fiber elongation*.

**Figure 4:**
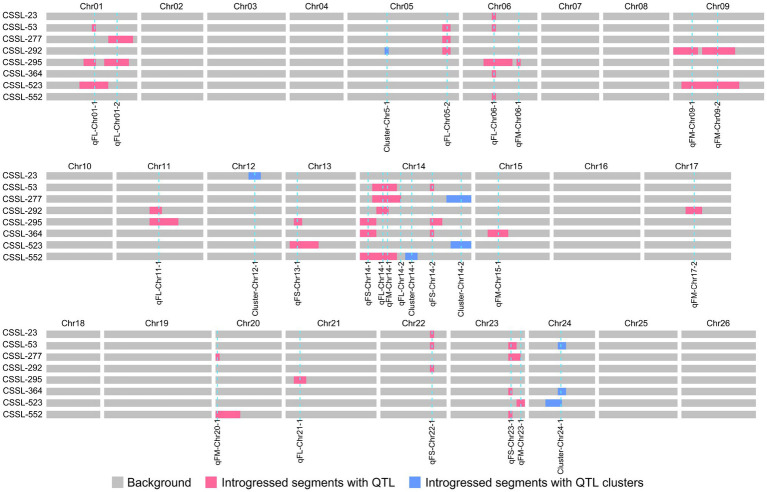
Distribution of chromosome introgressed segments for excellent lines.

The introgressed segments in eight superior lines were evaluated for favorable QTL alleles (Figure 4). These lines contained 34 fiber quality QTL, whose favorable alleles were derived from Pima S-7. Seven stable QTL (*qFL-*Chr06-1, *qFL-Chr12-1*, *qFL-Chr14-1*, *qFL-Chr14-2*, q*FL-Chr14-3*, *qFL-Chr14-4*, and *qFL-Chr21-1*) for fiber length were detected in one-to-five excellent lines. Two stable QTL (*qFS-Chr14-3* and *qFS-Chr24-1*) for fiber strength were identified in two and three excellent lines, respectively. Two stable QTL (*qFM-Chr09-1* and *qFM-Chr14-1*) for fiber micronaire were identified in two and four excellent lines, respectively. Among these QTL, seven QTL (*qFL-Chr06-1*, *qFL-Chr14-1*, *qFL-Chr14-3*, *qFL-Chr14-4*, *qFS-Chr24-1*, *qFM-Chr09-1*, and *qFM-Chr14-1*) were reported in the previous studies (Wang et al., 2006; Zhang et al., 2016; Si et al., 2017; Ma et al., 2018b; Zhao et al., 2021). These superior lines were valuable for cotton MAS breeding.

### Origin of Favorable Alleles in QTL Clusters

Significant negative correlations are common between lint yield and fiber quality traits (Wang et al., 2019; Zhang et al., 2020), as shown by the origin of favorable alleles of LP-QTL and fiber quality QTL in QTL clusters. Eight QTL clusters harbored both LP-QTL and FL-QTL, five QTL clusters harbored both LP-QTL and FS-QTL, and four QTL clusters harbored both LP-QTL and FM-QTL. Among these QTL clusters, the origin of favorable alleles of all LP-QTL was different from that of fiber quality QTL. The origin of favorable alleles in QTL clusters indicates that genes in QTL clusters may be closely linked or are pleiotropy and explains the significant phenotypic correlation between relevant traits and linkage drag (Wang et al., 2019; Zhang et al., 2020). It needs to further study whether the QTL clusters are caused by linkage or pleiotropy.

### WGCNA Provides a Powerful Approach to Screen Putative Candidate Genes in QTL Mapping

Several loci associated with yield and fiber quality traits have been identified through QTL mapping in cotton. However, it is challenging to identify a large number of genes using the traditional gene fine-mapping strategy due to the interval size of the identified QTL and the size of segregation population. Functionally related genes have similar expression profiles in related biological processes. Currently, large amount of transcriptome data can be used to reveal the related genes of certain biological processes based on the transcriptional coordination (co-expressed) among genes (Ruprecht et al., 2017). WGCNA is a technique used to explore the correlation patterns between genes and provides information on gene networks rather than individual genes (Langfelder and Horvath, 2008). Hub genes identified by WGCNA play related functions. For example, two homologous candidate genes (*Gh4CL4*) acting on green pigment biosynthesis were identified by WGCNA in the gene module associated with accumulation of green pigments (Sun et al., 2019). Moreover, two candidate genes (*Gh_D01G0162* and *Gh_D07G0463*) associated with increased LP were identified through GO enrichment and WGCNA analysis (Chen et al., 2021). In the present study, candidate genes located within the QTL intervals were evaluated rather than genes from the entire genome, which reduces background noise in gene co-expression studies. A total of 13 significant modules of genes associated with specific developmental stages and 586 putative candidate genes for 25 stable QTL were identified in the present study. These results provide important information for studying the mechanism of fiber quality formation.

### Candidate Genes for Fiber Quality QTL Are Related to the BR, ROS, and ETH Signaling Pathways

Two candidate genes encoding serine carboxypeptidase-like 40 (*SCPL40*, *GH_A08G1681*) and *probable receptor-like protein kinase PBL19* (*PBL19*, *GH_A12G2328*) were identified for *qFL-Chr08-2* and *qFL-Chr12-1*. *SCPL40* encodes a member of serine carboxypeptidase-like (*SCPL*) proteins, which plays important functions in plant growth and development, including processing proteins involved in brassinosteroid (BR) signaling pathway (Li et al., 2001). *PBL19* encodes a member of the receptor-like cytoplasmic kinase VII-4 (RLCK VII-4) subfamily (Bi et al., 2018) and some RLCK VII-4 gene family members are involved in the BR signaling pathway (Shi et al., 2013), which play key roles in cotton fiber elongation (Wang et al., 2020).

One candidate gene encoding heat shock protein-like 22.7 (*HSP22.7, GH_D02G0370*) was identified for *qFL-Chr14-1*. HSP22.7 protein is a member of the heat stress transcription factor (HSF) family. Members of the HSF family maintain homeostasis during fiber initiation and elongation (Sable et al., 2018). In addition, these proteins modulate reactive oxygen species (ROS) concentrations to regulate fiber development (Fan et al., 2021). Meanwhile, ROS is an important factor that regulates fiber cell tip growth (Qin and Zhu, 2011).

One candidate gene encoding GDSL esterase/lipase APG-like (*APG*, *GH_D05G1346*) was identified for *qFM-Chr19-1*. Several 19–25 DPA-specific genes are potentially regulated by *GhGDSL* in networks, including genes involved in cell wall and precursor synthesis (Yadav et al., 2017). In addition, the expression of GDSL members is regulated by ethylene (ETH) signaling components (Kim et al., 2013; Ma et al., 2018a).

## Conclusion

A total of 85 QTL for fiber quality and 20 QTL for lint percentage were identified across four environments in the CSSLs population. Thirteen significant modules of genes associated with specific developmental stages and 586 putative candidate genes for 25 stable QTL were identified through WGCNA. Furthermore, four candidate genes for stable QTL associated with fiber quality were identified. Eight superior lines with the best comprehensive phenotypes of LP, FL, FS, and FM in four environments were obtained in the present study. Moreover, the present study showed that *G. barbadense* and the excellent introgression lines had more favorable alleles for fiber quality QTL. These results provide valuable insight for breeding cotton cultivars with high yield and good fiber quality.

## Data Availability Statement

The datasets presented in this study can be found in online repositories. The names of the repository/repositories and accession number(s) can be found below: National Center for Biotechnology Information (NCBI) BioProject database under accession number PRJNA809429.

## Author Contributions

ZZ designed and supervised the experiments and contributed to the final editing of the manuscript, PY analyzed and summarized the data, generated the figures, and wrote the manuscript. PY, XS, XL, WW, YH, LC, JL, HH, TZ, WB, YT, XH, and MJ conducted field trials, phenotypic evaluation, and data collection. XS, XL, WW, and YH conducted DNA extraction and RNA-Seq analysis. KG and DL extracted RNA samples and performed qRT-PCR. ZT, DL, and JZ managed the CSSLs population. All authors read and approved the final manuscript.

## Funding

This work was funded by the National Natural Science Foundation of China (grant No. 31871670) and the National Key Research and Development Program of China (grant No. 2016YFD0100203-2).

## Conflict of Interest

The authors declare that the research was conducted in the absence of any commercial or financial relationships that could be construed as a potential conflict of interest.

## Publisher’s Note

All claims expressed in this article are solely those of the authors and do not necessarily represent those of their affiliated organizations, or those of the publisher, the editors and the reviewers. Any product that may be evaluated in this article, or claim that may be made by its manufacturer, is not guaranteed or endorsed by the publisher.

## Footnotes

^1^ http://www.geneontology.org/

^2^ https://www.kegg.jp/

^3^ http://www.omicsmart.com

## Supplementary Material

Supplementary Figure S1 Flow diagram showing the development process for the CSSLs population.

Supplementary Figure S2 Frequency distributions of phenotypic traits in the CSSLs population.

Supplementary Figure S3 Correlation analysis between the different traits in the same environments.

Supplementary Figure S4 Distribution of all QTL for the six traits on chromosomes.

Supplementary Figure S5 Global gene expression profile of CCRI35 and Pima S-7.

Supplementary Figure S6 Number of differentially expressed genes in CCRI35 and Pima S-7.

Supplementary Figure S7 Weighted gene co-expression network analysis (WGCNA) of candidate genes in CCRI35 and Pima S-7 at five time points of fiber development.

Supplementary Table S1 Phenotypic performance of lint percentage and fiber quality traits in the CSSLs.

Supplementary Table S2 Analysis of variation for lint percentage and fiber quality traits in the CSSLs.

Supplementary Table S3 Correlation coefficients among lint percentage and fiber quality traits in the CSSLs over the four environments.

Supplementary Table S4 Primers sequences used in the study.

Supplementary Table S5 Distribution of SSR markers on the 26 chromosomes.

Supplementary Table S6 Detailed information on the QTL for lint percentage and fiber quality traits in the CSSLs.

Supplementary Table S7 Summary of generated read data, quality control and mapping on the TM-1 genome for all samples.

Supplementary Table S8 Top 20 GO terms with significant enrichment of up- and down-regulated genes in different stages of fiber development.

Supplementary Table S9 Candidate genes within the QTL intervals.

Supplementary Table S10 GO annotation information of candidate genes in QTL for each trait.

Supplementary Table S11 KEGG annotation information of candidate genes in QTL for each trait.

Supplementary Table S12 Expression level of candidate genes in QTL clusters during fiber development.

Supplementary Table S13 GO annotation information of the candidate genes in QTL clusters.

Supplementary Table S14 KEEG annotation information of the candidate genes in QTL clusters.

Supplementary Table S15 Modules associated with specific stages of fiber development.

Supplementary Table S16 Putative candidate genes within the intervals of the stable QTL.

Supplementary Table S17 Primers sequences used in qRT-PCR.

Supplementary Table S18 SNP variation in the upstream and coding region of candidate genes.

Supplementary Table S19 Comparison of QTL identified in this study with those reported in previous studies.

Supplementary Table S20 Comparison of QTL identified in this study and previous GWAS results.
