# Supplementary material for: Identification of Candidate Genes for Lint Percentage and Fiber Quality Through QTL Mapping and Transcriptome Analysis in an Allotetraploid Interspecific Cotton CSSLs Population
Source: Front Plant Sci. 2022 Apr 29;13:882051. doi: 10.3389/fpls.2022.882051 (PMC9100888; doi:10.3389/fpls.2022.882051)
Supplement: Supplementary Figure S1 — Flow diagram showing the development process for the CSSLs population. [file Data_Sheet_1.PDF]

Identification of candidate genes for lint percentage and fiber quality through QTL mapping and transcriptome analysis in an allotetraploid interspecific cotton CSSLs population

Peng Yang, Xiaoting Sun, Xueying Liu, Wenwen Wang, Yongshui Hao, Lei Chen, Jun Liu, Hailun He, Taorui Zhang, Wanyu Bao, Yihua Tang, Xinran He, Mengya Ji, Kai Guo, Dexin Liu, Zhonghua Teng, Dajun Liu, Jian Zhang, Zhengsheng Zhang*

Supplementary Materials

Figures S1-S7

Tables S1-S3, S5, S7, S15, S17, S19, S20

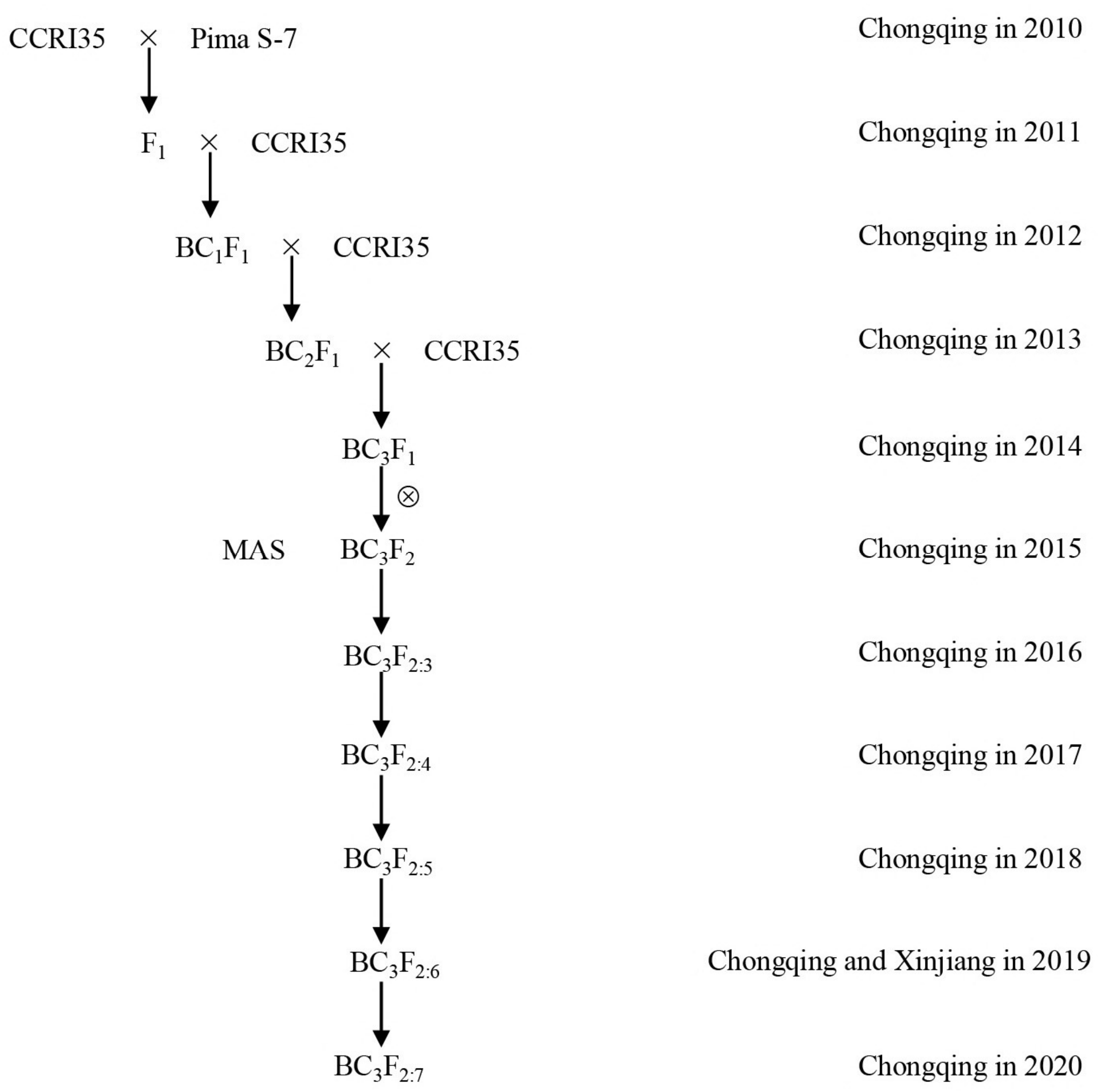

Figure S1. Flow diagram showing the development process for the CSSLs population.

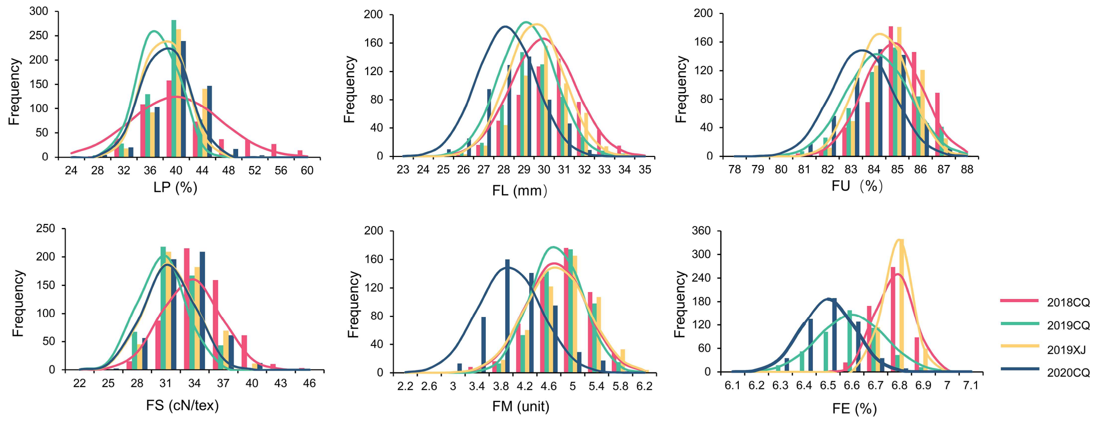

Figure S2. Frequency distributions of phenotypic traits in the CSSLs population.

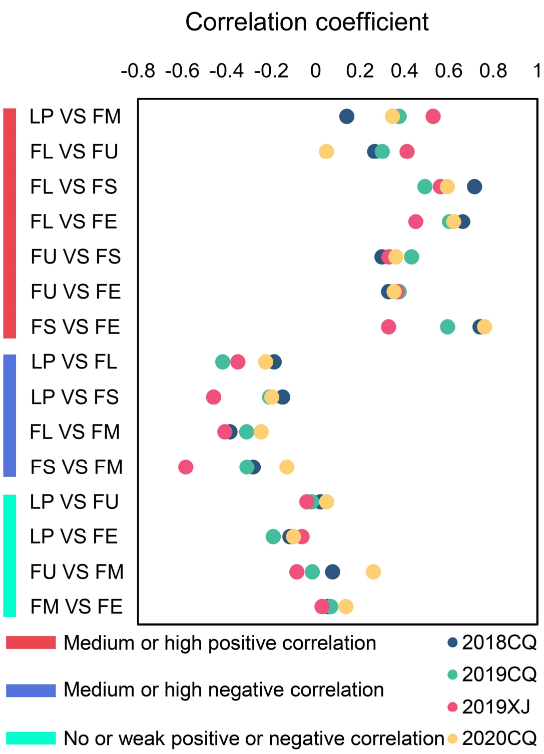

Figure S3. Correlation analysis between the different traits in the same environments.

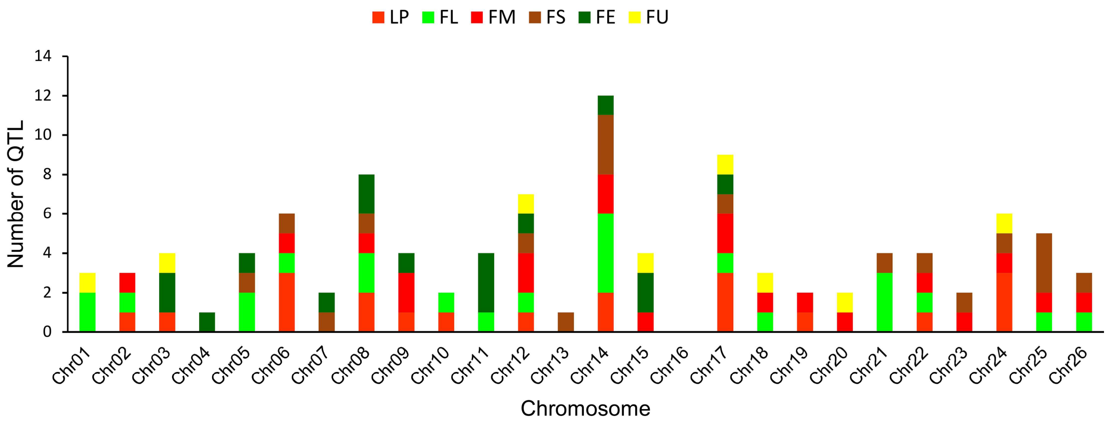

Figure S4. Distribution of all QTL for the six traits on chromosomes.

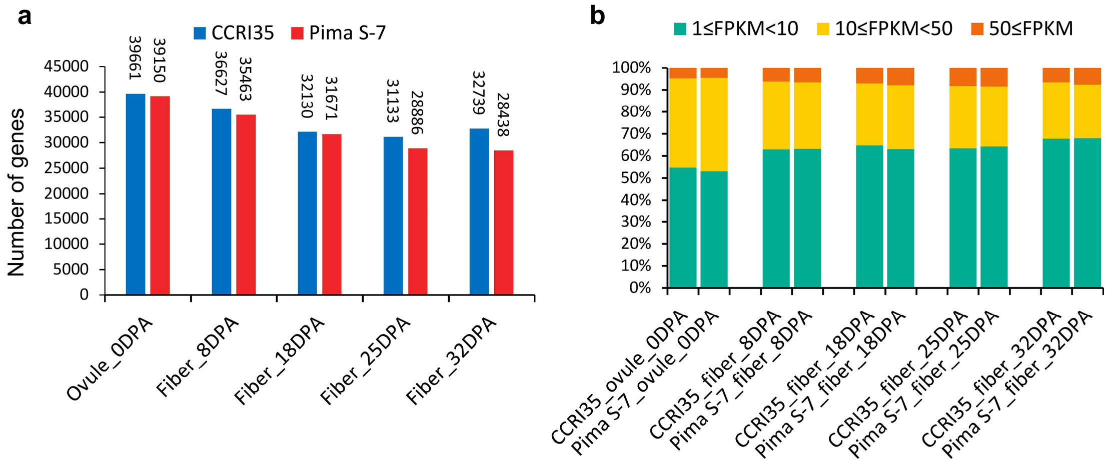

Figure S5. Global gene expression profile of CCRI35 and Pima S-7. a Total number of genes expressed in CCRI35 and Pima S-7during fiber development. **b** The fraction of genes expressed at different expressed levels.

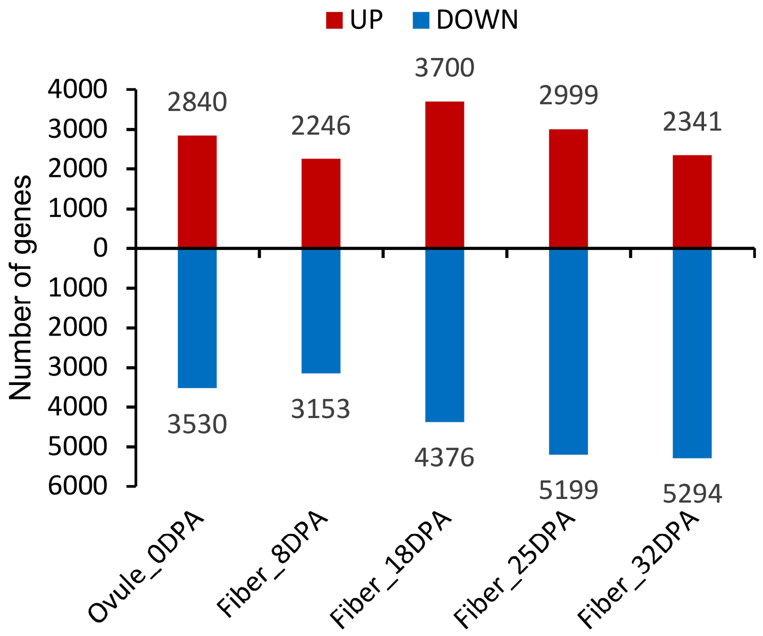

Figure S6. Number of differentially expressed genes in CCRI35 and Pima S-7.

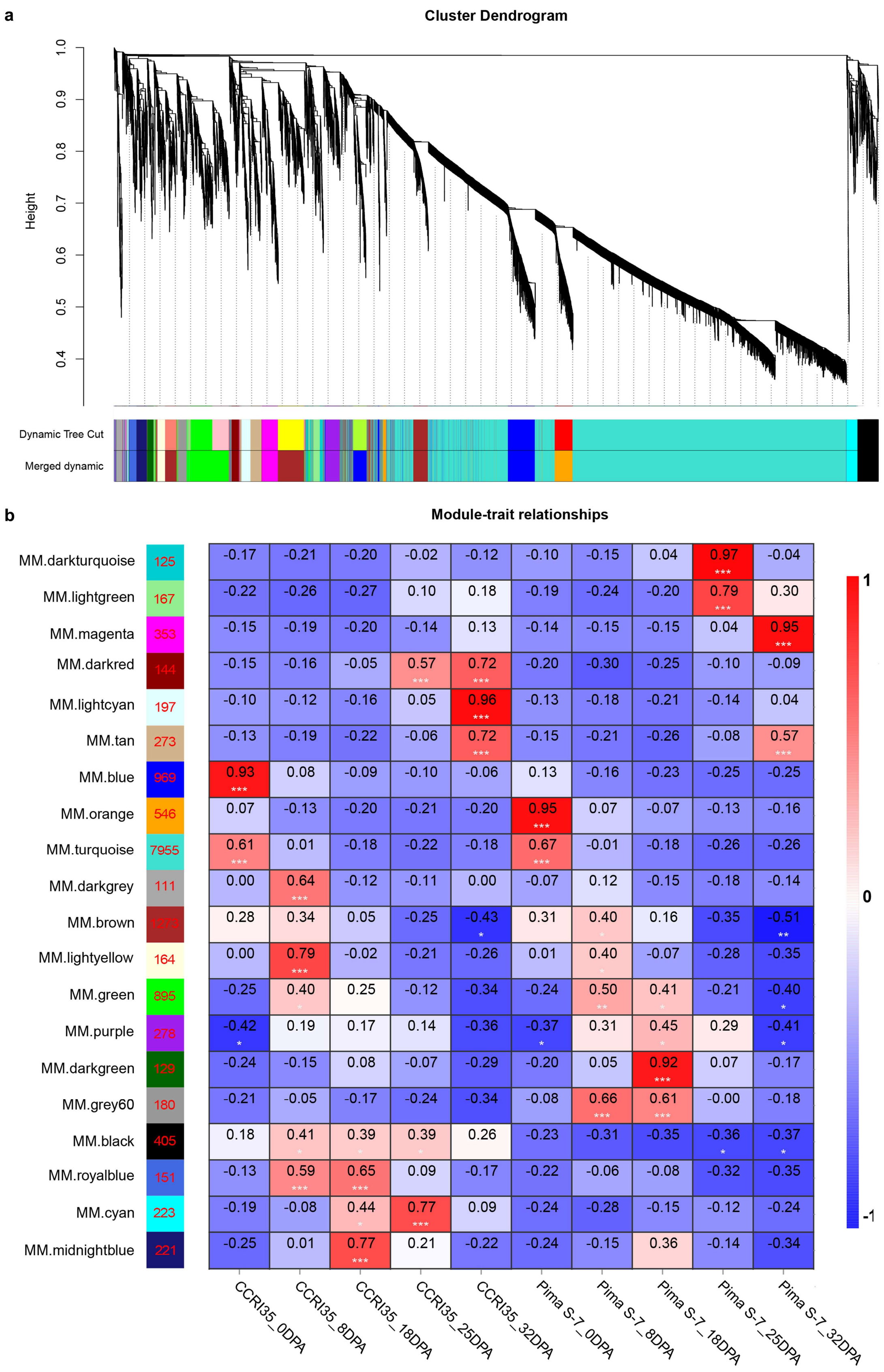

Figure S7. Weighted gene co-expression network analysis (WGCNA) of candidate genes in CCRI35 and Pima S-7 at five time points of fiber development. a Hierarchical dendrogram showing co-

expression modules identified by WGCNA. Each leaf in the tree represents one gene. The major tree was divided into 20 modules; each module is highlighted in a different color. **b** Module-trait relationships. Each row represents a module, and the correlation coefficient and significant levels are shown in each square. *, **, ***: significant levels of 0.5, 0.01, and 0.001, respectively. The number of genes in the module is shown in the left module squares, respectively.

Table S1 Phenotypic performance of lint percentage and fiber quality traits in the CSSLs.

Trait[a]	Environment[b]	CCRI35	Population								
		Mean	Maximum	Minimum	Range	Mean	SD[c]	Skewness	Kurtosis	CV (%)[d]	TRORP (%)[e]
LP (%)	2018CQ	37.03	59.62	26.36	33.26	39.93	6.86	0.84	0.11	17.19	61.05
	2019CQ	39.26	45.51	25.04	20.47	37.23	3.10	-0.62	0.95	8.32	24.05
	2019XJ	42.86	48.62	24.07	24.55	38.16	3.19	-0.73	1.48	8.35	3.81
	2020CQ	40.06	49.95	26.53	23.42	38.30	3.54	-0.24	0.75	9.24	30.28
FL (mm)	2018CQ	30.50	34.50	24.10	10.40	29.94	1.60	-0.14	0.12	5.34	36.23
	2019CQ	29.50	34.30	23.90	10.40	29.16	1.40	0.26	0.88	4.80	36.93
	2019XJ	31.40	33.60	24.80	8.80	29.58	1.40	-0.07	0.42	4.73	9.46
	2020CQ	30.00	32.10	23.90	8.20	28.11	1.45	-0.05	-0.20	5.17	10.43
FU (%)	2018CQ	84.30	87.60	79.30	8.30	84.80	1.25	-0.61	0.60	1.48	66.30
	2019CQ	82.30	87.60	78.20	9.40	84.15	1.39	-0.38	0.41	1.66	90.02
	2019XJ	85.90	87.20	79.00	8.20	84.36	1.13	-0.60	0.85	1.34	6.56
	2020CQ	84.90	86.60	78.10	8.50	83.49	1.32	-0.45	0.29	1.58	11.55
FS (cN/tex)	2018CQ	32.70	45.50	22.90	22.60	33.60	3.09	0.21	0.60	9.21	61.23
	2019CQ	32.80	37.40	23.60	13.80	30.55	2.43	0.09	-0.10	7.96	15.97
	2019XJ	30.40	41.50	24.90	16.60	31.41	2.62	0.44	0.20	8.33	60.42
	2020CQ	30.50	41.50	22.80	18.70	31.35	2.66	0.30	0.51	8.48	62.20
FM (unit)	2018CQ	4.80	6.00	2.80	3.20	4.72	0.51	-0.39	0.26	10.75	41.67
	2019CQ	4.50	6.00	3.10	2.90	4.72	0.44	-0.31	0.44	9.28	67.47
	2019XJ	4.30	6.20	2.90	3.30	4.73	0.53	-0.37	0.37	11.12	77.41
	2020CQ	4.50	5.50	2.40	3.10	3.94	0.53	0.39	0.13	13.41	10.80
FE (%)	2018CQ	6.80	7.00	6.60	0.40	6.78	0.08	-0.07	-0.22	1.15	16.49
	2019CQ	6.70	7.10	6.20	0.90	6.60	0.14	0.09	0.24	2.08	11.78
	2019XJ	6.90	6.90	6.60	0.30	6.79	0.06	-0.25	0.45	0.89	11.39
	2020CQ	6.60	6.80	6.10	0.70	6.50	0.11	0.04	0.19	1.74	8.19

[a]LP, lint percentage; FL, fiber length; FU, fiber uniformity; FS, fiber strength; FM, fiber micronaire; FE, fiber elongation

[b]2018CQ, 2018 in Chongqing; 2019CQ, 2019 in Chongqing; 2019XJ, 2019 in Xinjiang; 2020CQ, 2020 in Chongqing

[c]SD, Standard deviation

[d]CV, coefficient of variation

[e]TRORP, transgressive rate over the recurrent parent

Table S2 Analysis of variation for lint percentage and fiber quality traits in the CSSLs.

Trait	Factor	Sum of squares	DF[a]	MS	F
LP	Environment	1318.92	3	439.64	31.663***
	Genotype	14335.631	415	34.544	2.488***
	Error	17286.535	1245	13.885	
FL	Environment	865.469	3	288.49	240.991***
	Genotype	2220.435	439	5.058	4.225***
	Error	1576.576	1317	1.197	
FU	Environment	397.369	3	132.456	94.067***
	Genotype	1081.774	439	2.464	1.75***
	Error	1854.476	1317	1.408	
FS	Environment	2262.525	3	754.175	157.911***
	Genotype	6581.697	439	14.992	3.139***
	Error	6289.908	1317	4.776	
FM	Environment	211.975	3	70.658	610.428***
	Genotype	287.024	439	0.654	5.648***
	Error	152.445	1317	0.116	
FE	Environment	26.441	3	8.814	1156.189***
	Genotype	7.982	439	0.018	2.385***
	Error	10.039	1317	0.008	

[a]Excluded missing data when analysis the variance.

*** Indicates significance at the 0.001 level.

Table S3 Correlation coefficients among lint percentage and fiber quality traits in the CSSLs over the four environments.

Trait	Environment	LP	FL	FU	FS	FM	FE
LP	2018CQ	1	-0.187**	0.022	-0.149**	0.141**	-0.116*
LP	2019CQ	1	-0.418**	-0.017	-0.207**	0.377**	-0.191**
LP	2019XJ	1	-0.351**	-0.039	-0.460**	0.529**	-0.061
LP	2020CQ	1	-0.225**	0.049	-0.197**	0.346**	-0.100*
FL	2018CQ	-0.187**	1	0.266**	0.716**	-0.386**	0.663**
FL	2019CQ	-0.418**	1	0.300**	0.493**	-0.311**	0.603**
FL	2019XJ	-0.351**	1	0.412**	0.562**	-0.409**	0.452**
FL	2020CQ	-0.225**	1	0.049	0.593**	-0.246**	0.622**
FU	2018CQ	0.022	0.266**	1	0.299**	0.076	0.329**
FU	2019CQ	-0.017	0.300**	1	0.433**	-0.015	0.376**
FU	2019XJ	-0.039	0.412**	1	0.330**	-0.084	0.367**
FU	2020CQ	0.049	0.049	1	0.362**	0.261**	0.353**
FS	2018CQ	-0.149**	0.716**	0.299**	1	-0.281**	0.741**
FS	2019CQ	-0.207**	0.493**	0.433**	1	-0.310**	0.595**
FS	2019XJ	-0.460**	0.562**	0.330**	1	-0.585**	0.329**
FS	2020CQ	-0.197**	0.593**	0.362**	1	-0.129**	0.761**
FM	2018CQ	0.141**	-0.386**	0.076	-0.281**	1	0.056
FM	2019CQ	0.377**	-0.311**	-0.015	-0.310**	1	0.069
FM	2019XJ	0.529**	-0.409**	-0.084	-0.585**	1	0.029
FM	2020CQ	0.346**	-0.246**	0.261**	-0.129**	1	0.136**
FE	2018CQ	-0.116*	0.663**	0.329**	0.741**	0.056	1
FE	2019CQ	-0.191**	0.603**	0.376**	0.595**	0.069	1
FE	2019XJ	-0.061	0.452**	0.367**	0.329**	0.029	1
FE	2020CQ	-0.100*	0.622**	0.353**	0.761**	0.136**	1

* and ** indicate significant level at $P < 0.05$ and $P < 0.01$, respectively.

Table S5 Distribution of SSR markers on the 26 chromosomes.

Chromosome	No. of markers	Map length (cM)	Average distance (cM)
Chr01	22	126.46	5.75
Chr02	15	106.76	7.12
Chr03	19	131.34	6.91
Chr04	13	96.77	7.44
Chr05	27	235.34	8.72
Chr06	18	125.96	7.00
Chr07	14	144.82	10.34
Chr08	16	144.06	9.00
Chr09	22	169.25	7.69
Chr10	16	138.03	8.63
Chr11	21	208.62	9.93
Chr12	18	145.94	8.11
Chr13	17	148.33	8.73
Chr14	27	141.81	5.25
Chr15	18	127.11	7.06
Chr16	21	140.51	6.69
Chr17	21	120.94	5.76
Chr18	13	111.72	8.59
Chr19	26	217.86	8.38
Chr20	16	134.59	8.41
Chr21	22	202.39	9.20
Chr22	16	126.79	7.92
Chr23	18	137.02	7.61
Chr24	16	150.95	9.43
Chr25	19	137.64	7.24
Chr26	18	162.6	9.03
Maximum	27	235.34	10.34
Minimum	13	96.77	5.25
Average	19	147.45	7.92
Total	489	3833.61	7.92

Table S7 Summary of generated read data, quality control and mapping on the TM-1 genome for all samples.

Sample	Replicate	CleanData(bp)	Q30(%)	Total reads	Total_Mapped(%)
CCRI35_ovule_0DPA	1	5690962325	5401927392 (94.92%)	38111004	37009741 (97.11%)
CCRI35_ovule_0DPA	2	8803243471	8327627099 (94.60%)	58963542	57876274 (98.16%)
CCRI35_ovule_0DPA	3	8031763756	7593501863 (94.54%)	53829012	52829285 (98.14%)
CCRI35_fiber_8DPA	1	8361782592	7849423946 (93.87%)	55985552	54777043 (97.84%)
CCRI35_fiber_8DPA	2	7602898903	7174172232 (94.36%)	50912480	49909729 (98.03%)
CCRI35_fiber_8DPA	3	8960816628	8474589945 (94.57%)	59952042	58862922 (98.18%)
CCRI35_fiber_18DPA	1	8693560838	8213931841 (94.48%)	58120018	57061946 (98.18%)
CCRI35_fiber_18DPA	2	8429128092	7971627611 (94.57%)	56490690	55332406 (97.95%)
CCRI35_fiber_18DPA	3	7858350391	7423993012 (94.47%)	52653102	51583329 (97.97%)
CCRI35_fiber_25DPA	1	7251679811	6863202280 (94.64%)	48507310	47484623 (97.89%)
CCRI35_fiber_25DPA	2	7567369648	7184772688 (94.94%)	50604382	49571937 (97.96%)
CCRI35_fiber_25DPA	3	7279831696	6879980938 (94.51%)	48675236	47615856 (97.82%)
CCRI35_fiber_32DPA	1	7595348302	7167045905 (94.36%)	50841370	49647308 (97.65%)
CCRI35_fiber_32DPA	2	8403207210	7926600447 (94.33%)	56476476	55192385 (97.73%)
CCRI35_fiber_32DPA	3	7367766062	6953099564 (94.37%)	49292572	48298878 (97.98%)
Pima S-7_ovule_0DPA	1	7090749742	6702422662 (94.52%)	47544182	45839630 (96.41%)
Pima S-7_ovule_0DPA	2	7434079330	7025282634 (94.50%)	49806162	47931761 (96.24%)
Pima S-7_ovule_0DPA	3	8227197731	7781126045 (94.58%)	55023430	52943643 (96.22%)
Pima S-7_fiber_8DPA	1	7825396520	7392970498 (94.47%)	52361226	50772488 (96.97%)
Pima S-7_fiber_8DPA	2	7857552688	7430596701 (94.57%)	52608762	51044580 (97.03%)
Pima S-7_fiber_8DPA	3	9001265990	8494491481 (94.37%)	60225552	58383355 (96.94%)
Pima S-7_fiber_18DPA	1	8704855919	8223745987 (94.47%)	58310516	56408209 (96.74%)
Pima S-7_fiber_18DPA	2	7414444988	7009905934 (94.54%)	49829664	48204917 (96.74%)
Pima S-7_fiber_18DPA	3	9079045447	8589908951 (94.61%)	60726866	58785709 (96.80%)
Pima S-7_fiber_25DPA	1	7607255097	7195875805 (94.59%)	50891084	49194778 (96.67%)
Pima S-7_fiber_25DPA	2	8032489718	7559396193 (94.11%)	53695662	51752701 (96.38%)
Pima S-7_fiber_25DPA	3	8298956843	7796999137 (93.95%)	55424816	53511019 (96.55%)
Pima S-7_fiber_32DPA	1	7507481614	7098881089 (94.56%)	50188314	48431138 (96.50%)
Pima S-7_fiber_32DPA	2	8232551708	7745765414 (94.09%)	55050860	52642438 (95.63%)
Pima S-7_fiber_32DPA	3	8389984633	7907868120 (94.25%)	56172306	53688264 (95.58%)

Table S15 Modules associated with specific stages of fiber development.

Module	Specific stage
MM.blue	CCRI35_0DPA
MM.lightyellow	CCRI35_8DPA
MM.midnightblue	CCRI35_18DPA
MM.cyan	CCRI35_25DPA
MM.lightcyan	CCRI35_32DPA
MM.royalblue	CCRI35_8-18DPA
MM.black	CCRI35_8-25DPA
MM.darkred	CCRI35_25-32DPA
MM.orange	Pima S-7_0DPA
MM.darkgreen	Pima S-7_18DPA
MM.darkturquoise	Pima S-7_25DPA
MM.magenta	Pima S-7_32DPA
MM.grey60	Pima S-8_18DPA

Table S17 Primers sequences used in qRT-PCR.

QTL	Gene ID	Forward (5' to 3')	Reverse (5' to 3')
qFL-Chr08-2	GH_A08G1681	ACTGATGAGGCAGGCCAGGATA	CGAAATTTGCCACGGAAGGCTC
qFL-Chr12-1	GH_A12G2328	AAGGAATGGCTTGCGGAGGTTC	ATCCCTCTTTCCCCGTCTACCG
qFL-Chr14-1	GH_D02G0370	GTCAACCCAAGCCAACAGCCTA	TCTGATCCTTTGGGGACGGTCA
qFM-Chr19-1	GH_D05G1346	CCAGACCAGTACGGCTCATT	GCAACCTAACGGTGGAAGTG
	GH_A11G2385 (GhActin7)	TTGCAGACCGTATGAGCAAG	ATCCTCCGATCCAGACACTG

Table S19 Comparison of QTL identified in this study with those reported in previous studies.

Trait	QTL	Previously QTL	Reference
LP	qLP-Chr03-1	qLP-TX114-A3-1	Feng et al., 2019
LP	qLP-Chr08-1	qGhLP-A08-3	Shen et al., 2019
LP	qLP-Chr12-1	qLP-chr12-1, qLP-A12-1	Liang et al., 2015; Si et al., 2017
LP	qLP-Chr14-1	qLP14.1	Liu et al., 2017
LP	qLP-Chr17-2	qLP-C17-4	Shi et al., 2015
LP	qLP-Chr24-1	qLP-C24-1	Deng et al., 2019
FL	qFL-Chr02-1	qFL-C2-2	Shi et al., 2019
FL	qFL-Chr05-1	qFL-TX34-A5-1	Feng et al., 2019
FL	qFL-Chr05-2	qFL05.3	Tang et al., 2015
FL	qFL-Chr08-1	qFL-A08-1	Wang et al., 2019
FL	qFL-Chr10-1	qFL10.1, qFL-c10-1	Shao et al., 2014; Wang et al., 2016
FL	qFL-Chr14-1	qFL-D2-1	Wang et al., 2006
FL	qFL-Chr14-4	qFL-Pop1-D2-1	Zhang et al., 2016
FL	qFL-Chr25-1	qFL-Chr25-1	Ma et al., 2020
FL	qFL-Chr26-1	qFL-D12_14	Diouf et al., 2018
FU	qFU-Chr20-1	qFU-TX34-D10-1	Feng et al., 2019
FS	qFS-Chr05-1	qFS-TX34-A5-1	Feng et al., 2019
FS	qFS-Chr06-1	qFS-06-1	Li et al., 2019a
FS	qFS-Chr07-1	qFS-chr.7, qFS-C7-2, qFS-07-1	Cao et al., 2015; Deng et al., 2019; Li et al., 2019a
FS	qFS-Chr14-1	qFS-D2-1	Wang et al., 2006
FS	qFS-Chr14-2	qFS14.1	Shao et al., 2014
FS	qFS-Chr23-1	qFS-C23-1	Sun et al., 2012
FS	qFS-Chr25-1	qFS-25-5, qFS-C25-1b, qFS-Chr25-5	Li et al., 2019b; Wang et al., 2012a; Zhang et al., 2015
FS	qFS-Chr26-1	qFS-D12_14	Diouf et al., 2018
FM	qFM-Chr14-1	qMIC-D2-2, qFMIC-D2-1	Si et al., 2017; Wang et al., 2006
FM	qFM-Chr15-1	qMIC-Pop1-D1-1	Zhang et al., 2016
FM	qFM-Chr17-1	qFM-C17-2	Shi et al., 2020
FM	qFM-Chr19-1	qFM-C19-2	Deng et al., 2019
FM	qFM-Chr23-1	qFM23.3	Tang et al., 2015
FM	qFM-Chr24-1	qFM-C24-2	Shi et al., 2019
FE	qFE-Chr03-1	qFE-A3-1	Wang et al., 2019
FE	qFE-Chr04-1	qFE-TX34-A4-1	Feng et al., 2019
FE	qFE-Chr05-1	qFE-TX34-A5-1	Feng et al., 2019
FE	qFE-Chr08-1	qFE08.2	Tang et al., 2015
FE	qFE-Chr11-2	qFE-A11-1	Wang et al., 2019
FE	qFE-Chr15-2	qFE-Chr15-1	Ma et al., 2020
FE	qFE-Chr17-1	qFE-D3-1	Wang et al., 2019

Table S20 Comparison of QTL identified in this study and previous GWAS results.

Trait	QTL	Physical distance interval (bp)	Associated loci position	Reference
LP	qLP-Chr17-2	18817992-40983985	D03:35858092	Fang et al., 2017
LP	qLP-Chr17-2	18817992-40983985	D03:35874307	Fang et al., 2017
LP	qLP-Chr17-2	18817992-40983985	D03:35874307	Fang et al., 2017
LP	qLP-Chr17-2	18817992-40983985	D03:35874307	Fang et al., 2017
LP	qLP-Chr17-2	18817992-40983985	D03:35868596	Fang et al., 2017
LP	qLP-Chr17-2	18817992-40983985	D03:35874307	Fang et al., 2017
LP	qLP-Chr24-1	2444306-5418183	D08:2927709	Fang et al., 2017
LP	qLP-Chr24-1	2444306-5418183	D08:3046525	Fang et al., 2017
LP	qLP-Chr24-1	2444306-5418183	D08:3049681	Fang et al., 2017
FM	qFM-Chr09-2	8340396-55137633	A09:50230359	Fang et al., 2017
FM	qFM-Chr24-1	53358393-60195684	D08:60159948	Fang et al., 2017
FE	qFE-Chr08-2	70769530-93506509	A08:80798603	Fang et al., 2017
LP	qLP-Chr17-2	18817992-40983985	D03_36045920	Ma et al., 2018b
FL	qFL-Chr01-2	92351486-96955509	A01_96195441	Ma et al., 2018b
FL	qFL-Chr06-1	27929902-96044066	A06_92235377	Ma et al., 2018b
FL	qFL-Chr06-1	27929902-96044066	A06_92235391	Ma et al., 2018b
FL	qFL-Chr08-2	70769530-93506509	A08_73608506	Ma et al., 2018b
FL	qFL-Chr08-2	70769530-93506509	A08_88365843	Ma et al., 2018b
FL	qFL-Chr08-2	70769530-93506509	A08_88509581	Ma et al., 2018b
FL	qFL-Chr08-2	70769530-93506509	A08_88923232	Ma et al., 2018b
FL	qFL-Chr08-2	70769530-93506509	A08_88940929	Ma et al., 2018b
FL	qFL-Chr08-2	70769530-93506509	A08_88946344	Ma et al., 2018b
FL	qFL-Chr10-1	96332517-100152128	A10_100002003	Ma et al., 2018b
FL	qFL-Chr10-1	96332517-100152128	A10_100009968	Ma et al., 2018b
FL	qFL-Chr10-1	96332517-100152128	A10_100010010	Ma et al., 2018b
FL	qFL-Chr10-1	96332517-100152128	A10_100010122	Ma et al., 2018b
FL	qFL-Chr10-1	96332517-100152128	A10_100010136	Ma et al., 2018b
FL	qFL-Chr10-1	96332517-100152128	A10_100010180	Ma et al., 2018b
FL	qFL-Chr10-1	96332517-100152128	A10_100010192	Ma et al., 2018b
FL	qFL-Chr10-1	96332517-100152128	A10_100036690	Ma et al., 2018b
FL	qFL-Chr10-1	96332517-100152128	A10_100036874	Ma et al., 2018b
FL	qFL-Chr10-1	96332517-100152128	A10_100037696	Ma et al., 2018b
FL	qFL-Chr10-1	96332517-100152128	A10_100040438	Ma et al., 2018b
FL	qFL-Chr10-1	96332517-100152128	A10_100044193	Ma et al., 2018b
FL	qFL-Chr10-1	96332517-100152128	A10_100045197	Ma et al., 2018b
FL	qFL-Chr10-1	96332517-100152128	A10_100049352	Ma et al., 2018b
FL	qFL-Chr10-1	96332517-100152128	A10_100050995	Ma et al., 2018b
FL	qFL-Chr10-1	96332517-100152128	A10_100051351	Ma et al., 2018b
FL	qFL-Chr10-1	96332517-100152128	A10_100051392	Ma et al., 2018b
FL	qFL-Chr10-1	96332517-100152128	A10_100051801	Ma et al., 2018b
FL	qFL-Chr10-1	96332517-100152128	A10_100051860	Ma et al., 2018b
FL	qFL-Chr10-1	96332517-100152128	A10_100058113	Ma et al., 2018b
FL	qFL-Chr10-1	96332517-100152128	A10_100058193	Ma et al., 2018b
FL	qFL-Chr10-1	96332517-100152128	A10_100058525	Ma et al., 2018b
FL	qFL-Chr22-1	428691-1958468	D04_731149	Ma et al., 2018b
FL	qFL-Chr22-1	428691-1958468	D04_755121	Ma et al., 2018b
FL	qFL-Chr22-1	428691-1958468	D04_755581	Ma et al., 2018b
FL	qFL-Chr22-1	428691-1958468	D04_756340	Ma et al., 2018b

FL	qFL-Chr22-1	428691-1958468	D04_756429	Ma et al., 2018b
FL	qFL-Chr22-1	428691-1958468	D04_763603	Ma et al., 2018b
FL	qFL-Chr22-1	428691-1958468	D04_781083	Ma et al., 2018b
FL	qFL-Chr22-1	428691-1958468	D04_786863	Ma et al., 2018b
FL	qFL-Chr22-1	428691-1958468	D04_799800	Ma et al., 2018b
FL	qFL-Chr22-1	428691-1958468	D04_799819	Ma et al., 2018b
FU	qFU-Chr18-1	36200063-53841514	D13_48407308	Ma et al., 2018b
FU	qFU-Chr18-1	36200063-53841514	D13_48407529	Ma et al., 2018b
FU	qFU-Chr18-1	36200063-53841514	D13_50150726	Ma et al., 2018b
FU	qFU-Chr24-1	10009857-53358393	D08_24801472	Ma et al., 2018b
FU	qFU-Chr24-1	10009857-53358393	D08_34535845	Ma et al., 2018b
FU	qFU-Chr24-1	10009857-53358393	D08_61565498	Ma et al., 2018b
FU	qFU-Chr24-1	10009857-53358393	D08_61576435	Ma et al., 2018b
FU	qFU-Chr24-1	10009857-53358393	D08_61817008	Ma et al., 2018b
FS	qFS-Chr07-1	4687145-17277511	A07_5069413	Ma et al., 2018b
FS	qFS-Chr07-1	4687145-17277511	A07_5967896	Ma et al., 2018b
FS	qFS-Chr08-1	5204889-93506509	A08_19029303	Ma et al., 2018b
FS	qFS-Chr08-1	5204889-93506509	A08_19029325	Ma et al., 2018b
FS	qFS-Chr08-1	5204889-93506509	A08_19029326	Ma et al., 2018b
FS	qFS-Chr08-1	5204889-93506509	A08_23180686	Ma et al., 2018b
FS	qFS-Chr08-1	5204889-93506509	A08_27317781	Ma et al., 2018b
FS	qFS-Chr08-1	5204889-93506509	A08_27347419	Ma et al., 2018b
FS	qFS-Chr08-1	5204889-93506509	A08_27347445	Ma et al., 2018b
FS	qFS-Chr08-1	5204889-93506509	A08_32848282	Ma et al., 2018b
FS	qFS-Chr08-1	5204889-93506509	A08_35415740	Ma et al., 2018b
FS	qFS-Chr08-1	5204889-93506509	A08_47017597	Ma et al., 2018b
FS	qFS-Chr08-1	5204889-93506509	A08_48956464	Ma et al., 2018b
FS	qFS-Chr08-1	5204889-93506509	A08_50151708	Ma et al., 2018b
FS	qFS-Chr08-1	5204889-93506509	A08_54108930	Ma et al., 2018b
FS	qFS-Chr08-1	5204889-93506509	A08_88898722	Ma et al., 2018b
FS	qFS-Chr08-1	5204889-93506509	A08_88940929	Ma et al., 2018b
FS	qFS-Chr08-1	5204889-93506509	A08_88946344	Ma et al., 2018b
FS	qFS-Chr24-1	6911881-44507338	D08_29977479	Ma et al., 2018b
FM	qFM-Chr02-1	3103043-7989367	A02_5040453	Ma et al., 2018b
FM	qFM-Chr09-1	6879985-8340396	A09_8108582	Ma et al., 2018b
FM	qFM-Chr18-1	5252983-53841514	D13_15645548	Ma et al., 2018b
FM	qFM-Chr18-1	5252983-53841514	D13_42994963	Ma et al., 2018b
FM	qFM-Chr19-1	10375461-15753506	D05_12683719	Ma et al., 2018b
FM	qFM-Chr19-1	10375461-15753506	D05_13941003	Ma et al., 2018b
FM	qFM-Chr19-1	10375461-15753506	D05_13955716	Ma et al., 2018b
FM	qFM-Chr24-1	53358393-60195684	D08_55253515	Ma et al., 2018b
FM	qFM-Chr24-1	53358393-60195684	D08_60003809	Ma et al., 2018b
FM	qFM-Chr24-1	53358393-60195684	D08_60126646	Ma et al., 2018b
FM	qFM-Chr25-1	62012361-63719250	D06_62811372	Ma et al., 2018b
FM	qFM-Chr25-1	62012361-63719250	D06_62818197	Ma et al., 2018b
FM	qFM-Chr25-1	62012361-63719250	D06_62841100	Ma et al., 2018b
FM	qFM-Chr25-1	62012361-63719250	D06_62841639	Ma et al., 2018b
FE	qFE-Chr07-1	17277511-45550169	A07_18217292	Ma et al., 2018b
FE	qFE-Chr07-1	17277511-45550169	A07_18217475	Ma et al., 2018b
FE	qFE-Chr14-1	809850-3064403	D02_900391	Ma et al., 2018b

FL	qFL-Chr05-1	12881626-17154643	Gbar_A05_16273942	Zhao et al., 2021
FL	qFL-Chr06-1	28409129-107169080	Gbar_A06_44275086	Zhao et al., 2021
FL	qFL-Chr14-3	9534194-51790058	Gbar_D02_29896133	Zhao et al., 2021
FL	qFL-Chr14-3	9534194-51790058	Gbar_D02_29896134	Zhao et al., 2021
FU	qFU-Chr24-1	10159909-53259992	Gbar_D08_10490068	Zhao et al., 2021
FU	qFU-Chr24-1	10159909-53259992	Gbar_D08_10921551	Zhao et al., 2021
FS	qFS-Chr07-1	5464217-17971169	Gbar_A07_10988625	Zhao et al., 2021
FS	qFS-Chr24-1	7055918-44755719	Gbar_D08_10367876	Zhao et al., 2021
FS	qFS-Chr24-1	7055918-44755719	Gbar_D08_36346452	Zhao et al., 2021
FS	qFS-Chr24-1	7055918-44755719	Gbar_D08_36355760	Zhao et al., 2021
FS	qFS-Chr24-1	7055918-44755719	Gbar_D08_36355839	Zhao et al., 2021
FS	qFS-Chr24-1	7055918-44755719	Gbar_D08_38691427	Zhao et al., 2021
FS	qFS-Chr24-1	7055918-44755719	Gbar_D08_39206817	Zhao et al., 2021
FS	qFS-Chr24-1	7055918-44755719	Gbar_D08_39209928	Zhao et al., 2021
FS	qFS-Chr24-1	7055918-44755719	Gbar_D08_39272664	Zhao et al., 2021
FS	qFS-Chr26-1	46945964-52073417	Gbar_D12_49087075	Zhao et al., 2021
FS	qFS-Chr26-1	46945964-52073417	Gbar_D12_49087094	Zhao et al., 2021
FM	qFM-Chr06-1	110233736-113207610	Gbar_A06_111302117	Zhao et al., 2021
FM	qFM-Chr06-1	110233736-113207610	Gbar_A06_111302207	Zhao et al., 2021
FM	qFM-Chr06-1	110233736-113207610	Gbar_A06_111302824	Zhao et al., 2021
FM	qFM-Chr09-2	8362759-56696869	Gbar_A09_37791007	Zhao et al., 2021
FE	qFE-Chr09-1	70940284-74593320	Gbar_A09_72188791	Zhao et al., 2021
LP	qLP-Chr06-1	34666837- 115473485	A06:38376434	Ma et al., 2021
LP	qLP-Chr17-2	28577627-48889638	D03:43712431	Ma et al., 2021
LP	qLP-Chr17-2	28577627-48889638	D03:43721409	Ma et al., 2021
LP	qLP-Chr17-2	28577627-48889638	D03:43721468	Ma et al., 2021
LP	qLP-Chr17-2	28577627-48889638	D03:43726088	Ma et al., 2021
LP	qLP-Chr17-2	28577627-48889638	D03:43728033	Ma et al., 2021
LP	qLP-Chr17-2	28577627-48889638	D03:43729878	Ma et al., 2021
LP	qLP-Chr17-2	28577627-48889638	D03:43732059	Ma et al., 2021
LP	qLP-Chr17-2	28577627-48889638	D03:43818970	Ma et al., 2021
LP	qLP-Chr17-2	28577627-48889638	D03:43821322	Ma et al., 2021
FL	qFL-Chr10-1	113648659-117478533	A10:115624611	Ma et al., 2021
FS	qFS-Chr23-1	49402724-51964340	D09:49638569	Ma et al., 2021
